# *Agrobacterium tumefaciens* Small Lipoprotein Atu8019 Is Involved in Selective Outer Membrane Vesicle (OMV) Docking to Bacterial Cells

**DOI:** 10.3389/fmicb.2020.01228

**Published:** 2020-06-09

**Authors:** Lisa Roxanne Knoke, Sara Abad Herrera, Katrin Götz, Bo Højen Justesen, Thomas Günther Pomorski, Christiane Fritz, Sina Schäkermann, Julia Elisabeth Bandow, Meriyem Aktas

**Affiliations:** ^1^Faculty of Biology and Biotechnology, Department of Microbial Biology, Ruhr University Bochum, Bochum, Germany; ^2^Faculty of Chemistry and Biochemistry, Department of Molecular Biochemistry, Ruhr University Bochum, Bochum, Germany; ^3^Faculty of Biology and Biotechnology, Department of Applied Microbiology, Ruhr University Bochum, Bochum, Germany

**Keywords:** outer membrane vesicles, lipoprotein, small protein, surface protein, Atu8019, entericidin, cell attachment, *Agrobacterium tumefaciens*

## Abstract

Outer membrane vesicles (OMVs), released from Gram-negative bacteria, have been attributed to intra- and interspecies communication and pathogenicity in diverse bacteria. OMVs carry various components including genetic material, toxins, signaling molecules, or proteins. Although the molecular mechanism(s) of cargo delivery is not fully understood, recent studies showed that transfer of the OMV content to surrounding cells is mediated by selective interactions. Here, we show that the phytopathogen *Agrobacterium tumefaciens*, the causative agent of crown gall disease, releases OMVs, which attach to the cell surface of various Gram-negative bacteria. The OMVs contain the conserved small lipoprotein Atu8019. An *atu8019*-deletion mutant produced wildtype-like amounts of OMVs with a subtle but reproducible reduction in cell-attachment. Otherwise, loss of *atu8019* did not alter growth, susceptibility against cations or antibiotics, attachment to plant cells, virulence, motility, or biofilm formation. In contrast, overproduction of Atu8019 in *A. tumefaciens* triggered cell aggregation and biofilm formation. Localization studies revealed that Atu8019 is surface exposed in *Agrobacterium* cells and in OMVs supporting a role in cell adhesion. Purified Atu8019 protein reconstituted into liposomes interacted with model membranes and with the surface of several Gram-negative bacteria. Collectively, our data suggest that the small lipoprotein Atu8019 is involved in OMV docking to specific bacteria.

## Introduction

The release of membrane vesicles (MVs) is ubiquitous among bacteria. MVs from Gram-negative bacteria are usually termed outer membrane vesicles (OMVs). They can be formed by budding off the outer membrane (OM) after cargo enclosure by various mechanisms (Hoekstra et al., 1976; Zhou et al., 1998; Lee et al., 2008; Bonnington and Kuehn, 2014; Kim et al., 2015; Schwechheimer and Kuehn, 2015; Toyofuku et al., 2017; Toyofuku, 2019). These spherical bilayered particles (20–400 nm) are composed of lipopolysaccharides (LPS), peptidoglycan, phospholipids, and proteins. Remarkably, the lumen of OMVs carries a cocktail of diverse molecules including lytic enzymes, antimicrobial compounds, hydrophobic signal molecules, and genetic material (Renelli et al., 2004; Koeppen et al., 2016). MVs have multiple functions, which are determined by their cargo molecules. They promote bacterial fitness and survival, especially in mixed communities (Guerrero-Mandujano et al., 2017). As lipid-enclosed particles, they serve as delivery vehicles over long distances and time periods protecting a variety of biomolecules by increasing their extracellular stability. Hence, they play important roles in bacterial communication with the environment and surrounding (micro-) organisms. They contribute to highly communicative processes like biofilm formation, bacterial competition, quorum sensing, and DNA/RNA-transfer in bacterial communities (Kulp and Kuehn, 2010; Park et al., 2014; Kaparakis-Liaskos and Ferrero, 2015; Sjöström et al., 2015; Wang et al., 2015). In pathogenic bacteria, MVs often play important roles in host colonization, modulation of host immunity, and pathogenicity (Kuehn and Kesty, 2005; Ionescu et al., 2014; O’Donoghue and Krachler, 2016). Internalization of MVs by mammalian host cells is well-studied and different mechanisms have been described. MVs can enter eukaryotic cells by clathrin or caveolin-dependent endocytosis, via lipid-raft domains, or by direct membrane fusion (Kaparakis et al., 2010; Berleman and Auer, 2013; O’Donoghue and Krachler, 2016; Tashiro et al., 2017; Nagakubo et al., 2019). Although transfer of MV’s cargo into target bacteria has been shown in many studies, content uptake by bacterial cells is poorly understood (Klieve et al., 2005; Mashburn and Whiteley, 2005; Tashiro et al., 2017; Toyofuku et al., 2017; Nagakubo et al., 2019). It has been proposed that MVs are lysed after target cell contact and the content (DNA) is taken up by Type IV pilus-mediated transport (Fulsundar et al., 2014). Other studies suggested uptake of MV content by membrane fusion (Kadurugamuwa and Beveridge, 1996; Tashiro et al., 2017; Nagakubo et al., 2019). Recent studies provided evidence that MVs selectively interact with bacteria in order to transfer their content to the target cells (MacDonald and Beveridge, 2002; Lin et al., 2017; Tashiro et al., 2017; Brameyer et al., 2018). However, the underlying mechanism(s) of specific vesicle docking and internalization by target bacteria remains poorly understood. As recently shown, MVs from the Gram-negative enterobacterium *Buttiauxella agrestis* specifically interact with bacteria from the same genus. For this selective interaction, special physiochemical cell-surface properties of *B. agrestis* were discussed. Since *B. agrestis* MV-cell attachment is reduced by proteinase K treatment, the selective interaction seems to require yet unknown surface-exposed proteins (Tashiro et al., 2017; Tashiro et al., 2019). A specific ligand-receptor mediated interaction of MVs from *Pseudomonas aeruginosa* and *Vibrio harveyi* has been recently suggested. In *P. aeruginosa*, uptake of OMVs containing *Pseudomonas* quinolone (PQS)-Fe^3+^ is proposed to be mediated by the OMV-associated TseF protein and the cell-surface receptors FptA or OprF (Lin et al., 2017; Lin et al., 2018). The marine pathogen *V. harveyi* packages the quorum sensing (QS) molecule CAI-1 into OMVs, which trigger a QS phenotype in CAI-1 lacking *V. harveyi* and in *Vibrio cholerae* cells (Brameyer et al., 2018). Indeed, OMVs are enriched with surface-attached proteins, particularly lipoproteins (LPPs; Kaparakis-Liaskos and Ferrero, 2015; Valguarnera et al., 2018), which could mediate specific attachment and internalization of OMVs to target bacteria. Bacterial LPPs are involved in diverse physiological processes like cell shape maintenance, OM biogenesis, transport, motility, signal transduction, virulence, and stimulation of innate immune reactions by mammalian Toll-like receptors (Cascales et al., 2002; Kovacs-Simon et al., 2011; Nakayama et al., 2012; Narita and Tokuda, 2017; Burgess et al., 2018). LPPs are characterized by their N-terminal signal peptidase II sequence and a “lipobox” [(LVI) (AST VI) (GAS) (C)]. The lipobox within the N-terminal signal sequence targets the protein for lipid modification. The highly conserved cysteine residue within the lipobox is crucial for the acylation of LPPs (Hantke and Braun, 1973; Inouye et al., 1977; Nakayama et al., 2012; Sutcliffe et al., 2012). The prepro-LPP is synthesized in the cytoplasm and translocated to the periplasmic site of the inner membrane where the modifications can take place. An S-diacylglyceroltransferase diacylates the thiol group of the conserved cysteine within the lipobox. This enables the signal peptidase II to hydrolyse the signal peptide and to form the mature diacylated LPP. Depending on protein and organism, an N-acyltransferase can acylate the newly generated α-amino group to produce a triacylated LPP. The fatty acids attached to LPPs are derived from phospholipids and offer a powerful regulatory tool to modulate localization and function (Sankaran and Wu, 1994; Nakayama et al., 2012; Buddelmeijer, 2015; Vogeley et al., 2016). Depending on the N-acylation and specific sorting signals, LPPs either remain in the inner membrane or they are translocated to the inner leaflet of the OM by the LPP OM-localization pathway (Lol; Zückert, 2014; Goolab et al., 2015; Kaplan et al., 2018). Once located in the OM, LPPs either remain at the periplasmic site or they are transported to the cell surface. The underlying mechanism(s) of LPP transport across the OM is not fully understood. Different mechanisms have been proposed for surface exposure of LPPs like the involvement of Type II or Type V secretion systems, the beta-barrel assembly machinery (BAM), and species-specific lipid transporters. Some LPPs are surface-exposed even in heterologous host cells, suggesting an additional, and yet unidentified universally conserved mechanism (Arnold et al., 2014; Zückert, 2014; Konovalova and Silhavy, 2015; Hooda et al., 2016; Wilson and Bernstein, 2016; Fantappie et al., 2017).

In the present study, we investigated OMV formation and the functional role of a small lipoprotein (Atu8019), which we found in OMVs from the phytopathogen *Agrobacterium tumefaciens*. *A. tumefaciens* is the causative agent of crown-gall disease and routinely used as vehicle for plant transformation (Goodner et al., 2001; Wood et al., 2001; Escobar and Dandekar, 2003). If and how OMVs play a role in *A. tumefaciens* physiology and virulence has never been investigated.

## Materials and Methods

### Strains, Plasmids, and Growth Conditions

Bacterial strains and plasmids used in this study are listed in Supplementary Table S1. *Escherichia coli* JM83 served as host for plasmid construction and storage. The BL21 (DE3) strain was used for recombinant protein production using the pET-expression system. *E. coli* strains were routinely cultivated in Luria-Bertani (LB) medium with respective antibiotics if required (ampicillin 100 μg/mL or kanamycin 50 μg/mL) at 37°C. BL21-expression cultures containing pET-derivatives were induced with 0.4 mM IPTG at an optical density (OD) of ∼0.6–0.8 and cultivated for 4 h at 30°C. *A. tumefaciens* C58 wildtype and derivatives were routinely cultivated in LB medium at 30°C. *A. tumefaciens* overexpression strains containing pTrc-derivatives were cultivated in LB medium containing 100 μg/mL streptomycin and 300 μg/mL spectinomycin. Protein expression was induced with 0.4 mM IPTG when the cultures reached an OD_600_ of ∼0.6–0.8. Expression cultures were incubated for 18 h at 30°C after induction, if not stated differently. Target bacteria *E. coli* DH10B and *Bacillus subtilis* were cultivated in LB medium at 37°C. *Xanthomonas campestris* and *Pseudomonas syringae* were grown in LB medium at 30°C and *Sinorhizobium meliloti* was cultivated in TY-medium (0.5% tryptone, 0.3% yeast extract, and 0.07% CaCl_2_) at 30°C.

### Construction of Expression Vectors to Produce HIS-Tagged Proteins

The *atu8019* gene was purchased from Eurofins Genomic (Würzburg) in a pEX-A2 vector (pEX_8019_syn) flanked by NdeI and XhoI restriction sites. The gene was restricted by NdeI and XhoI and cloned into the pET24b expression vector. The resulting plasmid (pBO6126) encodes a C-terminally HIS-tagged Atu8019-fusion protein.

For homologous overproduction of C-terminally HIS-tagged Atu8019-fusion proteins, we used the broad host IPTG-inducible expression vector pTrc200 (Schmidt-Eisenlohr et al., 1999). The plasmid pBO6126 served as DNA template for PCR amplification of the *atu8019* gene including the 3’prime HIS-coding sequence (primers are listed in Supplementary Table S2). The PCR product was cloned into pTrc200 with appropriate enzyme sites, resulting in the plasmid pBO6128 (pTrc-atu8019-HIS).

To construct a plasmid for recombinant Atu2451 production, the corresponding gene was PCR-amplified from chromosomal DNA with appropriate primers (Supplementary Table S2). The PCR product was cloned via the restriction sites NdeI and XhoI into pET24b resulting in the plasmid pBO1920 encoding the Atu2451 protein with a HIS-tag at the C-terminus.

### Construction of the Atu8019C22A^HIS^ Variant

To create an Atu8019C22A variant, site-directed mutagenesis was performed using the QuikChange mutagenesis kit (Agilent, Waldbronn, Germany) according to manufacturer’s protocol. The QuikChange-PCR was performed with pBO6128 (pTrc-derivative) or pBO6126 (pET-derivative) as template and appropriate oligonucleotides (Supplementary Table S2). The resulting plasmids pBO6104 (pTrc-atu8019C22A) and pBO6127 (pET-atu8019C22A) encode Atu8019C22A variants carrying a C-terminal HIS-tag.

### Construction of a Markerless *A. tumefaciens atu8019*-Deletion Mutant

The markerless *atu8019*-deletion strain was constructed as described before (Wessel et al., 2006) using the suicide plasmid pK19mobsacB and appropriate primers listed in Supplementary Table S2. PCR fragments corresponding to upstream (517 bp) and downstream (422 bp) regions of the *atu8019* gene were cloned with appropriate enzyme sites into pK19mobsacB. The resulting plasmid (pBO6100) was transferred into *A. tumefaciens* and potential deletion mutants, indicated by sucrose resistance and kanamycin sensitivity, were confirmed by Southern blot analysis.

### Construction of *A. tumefaciens atu8019*^FLAG^ Reporter Strain and Epitope Tagging

The plasmid for the *atu8019*^FLAG^ reporter strain construction was cloned according to (Hoffmann et al., 2015). Primer pairs used to PCR amplify the *A. tumefaciens atu8019* gene are listed in Supplementary Table S2. The amplification product, in which the stop codon was exchanged against three cytidine residues (CCC) was blunt-end cloned into the SmaI site of pYP168. The SmaI site downstream of the *atu8019*-coding region was recovered by the CCC extension and was used to insert the SmaI fragment carrying the FLAG-KaT cassette from plasmid pYP247. The resulting pBO6102 (*atu8019*^FLAG^) hybrid plasmid was transferred by electroporation into *A. tumefaciens*. *Agrobacterium* strains, which integrated the plasmid into their chromosome by single homologous recombination were selected by kanamycin resistance. The resulting *A. tumefaci*ens: pBO6102 (*atu8019*^FLAG^), strain was examined for Atu8019^FLAG^ production by Western blot analysis using FLAG-epitope-specific antibodies.

All plasmids constructed in this study were verified by DNA sequencing.

### Subcellular Fractionation and Protein Localization

For Atu8019 localization studies, *A. tumefaciens atu8019*^FLAG^ reporter strain was cultivated in LB medium (kanamycin, 50 μg/mL) to an OD_600_ of 1.0. For Atu2451 localization, an *E. coli* BL21 expression culture carrying pBO1920 was prepared. Cells were harvested and the culture supernatants were used for OMV isolation as described below. The cell pellet was resuspended in lysis buffer (50 mM Tris, 100 mM NaCl, and pH 8.0) and treated with a spatula tip of lysozyme, DNaseI, and PMSF for 30 min on ice. Cells were disrupted using a French pressure cell (SLM Instruments; 3 × 900 kpsi). After cell lysis, debris was removed by centrifugation (15,000 × *g*, 4°C, and 20 min) and the cell lysates were fractionated into cytosol and membrane-fractions by high-speed centrifugation (200,000 × *g*, 4°C, and 1.5 h). The cytosol fractions were stored at -20°C and membranes were further fractionated into inner and outer membranes by *N*-lauroylsarcosine extraction. This detergent dissolves selectively inner membranes, leaving outer membranes intact (Hobb et al., 2009). Membrane pellets were solubilized in 0.5% *N*-lauroylsarcosine in lysis buffer for 12 h at 4°C and inner and outer membranes were separated by high-speed centrifugation (100,000 × *g*, 1 h, and 4°C). Outer membrane pellets were dissolved in lysis buffer containing 0.5% Triton X-100. Proteins from the corresponding fractions were precipitated using TCA (trichloroacetic acid) and resolved in SDS sample buffer. For protein detection, 50 μg of protein from each fraction was loaded onto an SDS gel and specific proteins were detected by Western blot analysis.

### Immunofluorescence Staining

*Agrobacterium tumefaciens* FLAG reporter strains were cultivated in LB medium until stationary phase. Cells were harvested from 2 mL cultures, washed three times with TBS buffer (25 mM Tris, 150 mM NaCl, and pH 7.6), and suspended in 1 mL TBS buffer to a final OD_600_ of 2.0. Cells were fixed with 162.5 μL paraformaldehyde (16%, v/v) and 11.7 μL glutaraldehyde (1%, v/v) for 15 min at RT and then washed with TBS buffer (3 × 1 mL, 10,000 × *g*; RT) followed by washing with 1 mL GTE-buffer (50 mM glucose, 20 mM Tris, and 10 mM EDTA). For cell wall permeabilization, 5 mg/mL lysozyme in 5 mM EDTA (dissolved in water) was added. After 5 min on ice, cells were washed with 1 mL TBS buffer and incubated for 5 min at RT in TBS buffer containing 0.1% Triton X-100 (TBST). Blocking was performed with TBST buffer containing 3% (w/v) BSA for 1 h at RT. After a washing step with TBS, FLAG-tagged proteins were labeled by incubating the samples with an anti-FLAG antibody [1:70 in TBST containing 0.1% (w/v) BSA] for 1 h at 30°C. AlexaFluor488 coupled goat-anti-mouse antibody was used at 1:250 as secondary antibody. For fluorescence microscopy cells were resuspended in 200 μL TBS buffer and 2 μL were spotted onto a microscopy slide covered with agarose [1.5% (w/v) in TBS].

### Outer Membrane Vesicle Isolation

For OMV isolation from *A. tumefaciens* wildtype and derivatives, strains were cultivated in LB medium to an OD_600_ of 1.0 if not stated otherwise. For OMV isolation from *E. coli*, we used 4 h expression cultures carrying the appropriate expression plasmid. Cells were harvested at 15,000 × *g* for 10 min at 4°C. The culture supernatants were filtered (0.2 μm) and the cell-free filtrates were fractionated into OMV-associated and secreted soluble proteins by high-speed centrifugation (150,000 × *g*, 4 h, and 4°C). The OMV pellet isolated from 100 mL culture supernatant, was resuspended in 1 mL 10 mM KH_2_PO_4_ (pH 7.4) if not stated differently. Sterility of samples was assessed on LB-agar plates. The supernatant containing secreted soluble proteins was precipitated with TCA and 50 μg of proteins from each fraction were analyzed by SDS-PAGE and Western blot.

### Proteinase Accessibility Assays With Cells and OMVs

Surface exposure of Atu8019 in *A. tumefaciens* cells and OMVs was analyzed by proteinase-accessibility assays. Briefly, the *A. tumefaciens atu8019*^FLAG^ reporter strain was cultivated to stationary phase in 50 mL LB medium (kanamycin 50 μg/mL). The culture was split in two aliquots and centrifuged (1.500 × *g*, 10 min, and 4°C). Cell pellets were resuspended in 3.5 mL Tris buffer (50 mM Tris, 7.5 mM CaCl_2_, and pH 8.8). One of the samples was lysed using French press and the debris was removed by centrifugation (15.000 × *g*, 20 min, and 4°C). Aliquots of the lysed and intact cells were incubated with increasing amounts of proteinase K (0, 10, 25, and 100 μg/mL) and incubated for 30 min at RT. The reaction was stopped by addition of 20 μL of PMSF (100 mM in isopropanol), followed by 5 min incubation at RT. Samples were boiled for 5 min at 97°C in SDS sample buffer and equal amounts were loaded onto a 12.5% SDS gel for protein visualization and Western blot analysis.

To test display of Atu8019 in the context of OMVs, proteinase-accessibility assays were performed with OMVs derived from the *atu8019*^FLAG^ reporter strain. Equal amounts of OMV aliquots were incubated in PBS buffer (137 mM NaCl, 2.7 mM KCl, 10 mM Na_2_HPO_4_, 2 mM KH_2_PO_4_, and pH 7.5) with 0.5 μg/mL proteinase K in the absence or presence of 1% SDS to disrupt the vesicles. An untreated OMV sample and an OMV-reaction mixture containing SDS, proteinase K, and 2 mM PMSF were included as controls. Samples were incubated for 30 min at 37°C before reaction was stopped by addition of 2 mM PMSF. Atu8019^FLAG^ was detected by Dot blot analysis.

### Transmission Electron Microscopy of OMVs

For visualization by transmission electron microscopy, a 10 μL aliquot of isolated OMVs was negatively stained with 1% uranyl acetate for 7 min at RT. Samples were spotted onto a copper grid and analyzed using the Philips 420 Transmission Electron Microscope equipped with a Gatan digital camera. To estimate the size of OMVs, 10 nm sized gold particles were used for the appropriate magnification.

### Dynamic Light Scattering Analysis of OMVs

Isolated OMVs were diluted 1:100 in PBS buffer and vesicle size was determined by dynamic light scattering (Particle sizer, Malvern instruments). Data was collected from 1 mL of PBS buffer suspended OMVs by 15 acquisitions at 25°C.

### SDS-PAGE, Western Blot and Dot Blot Analyses

Protein samples from cellular or subcellular fractions were precipitated using TCA and subjected to standard SDS-PAGE followed by coomassie brilliant blue staining. For Dot blot, samples (4 μl) were spotted onto a nitrocellulose membrane (GE healthcare). Western blot analyses were carried out by standard methods using specific antibodies: For RpoA, FtsH, and OmpA detection, first antibodies specific for the respective protein (1:5,000), and an anti-rabbit HRP conjugate were used (1:5,000). For FLAG-tagged proteins, we used a FLAG-specific first antibody (1:4,000), and an anti-mouse HRP conjugate (1:5,000). HIS-tagged proteins were detected using a penta-HIS-HRP conjugate (1:4,000). Proteins were detected by chemiluminescence using the FluorChemTM SP Multiimager (AlphaInnotech).

### Mass Spectrometry Analysis

For MS analysis, we isolated OMVs from *A. tumefaciens* WT cells cultivated in LB medium to an OD_600_ of 1.0. OMV proteins were precipitated using TCA and concentration was determined in Tris buffer (10 mM Tris–HCl, and pH 8.0) by IR spectrometry (DirectDetect^®^ Infrared spectrometer, Merck Millipore). Samples for replicates 1, 2 and 3 contained 0.99, 1.69, and 1.45 μg/μL protein, respectively. 8 μL of each samples were loaded onto a 14% SDS-gel. To reduce protein loss, gels were stopped directly after the samples entered the separating gel. Bands were excised from the gel and proteins were digested tryptically and subjected to LC-MS as described previously (Wenzel et al., 2013) with the following modifications. After tryptic digestion, peptides were eluted using 50% acetonitrile containing 0.1% formic acid. Samples for replicates 1, 2, and three were dried *in vacuo* and resuspended in 105.9, 180.5, and 155.1 μL 0.1% formic acid, respectively. 4 μL of each sample were subjected to LC-MS. Data were analyzed using the ProteinLynx Global Server (version 2.5.2; Waters) with a nonredundant *A. tumefaciens* database (BioProject PRJNA57865) containing 5,557 protein entries (including sequences for trypsin and keratin). Processing and workflow parameters were as follows: chromatographic peak width, automatic; MS TOF resolution, automatic; lock mass for charge 2, 556.2771 Da/e; lock mass window, 0.3 Da; low energy threshold, 50 counts; elevated energy threshold, 15 counts; intensity threshold, 500 counts; peptide tolerance, automatic; fragment tolerance, automatic; min fragment ion matches per peptide, 2; min fragment ion matches per protein, 2; min peptide matches per protein, 1; maximum protein mass, 250,000 Da; primary digest reagent, trypsin; secondary digest reagent, none; missed cleavages, 1; fixed modifications, carbamidomethyl C; variable modifications, deamidation N, deamidation Q, oxidation M; and false positive rate, 4.

### Lipid Analysis

Membrane lipids from cells or OMVs were isolated according to Bligh and Dyer (1959). For isolation of cellular lipids, an aliquot of 2 mL cells was harvested, washed, and resuspended in 100 μL water. For isolation of OMV lipids, we used 500 μL of OMV samples, isolated from the supernatant of 100 mL cultures. Lipids were spotted onto HPTLC silica 60 plates (Merck, Darmstadt, Germany) and separated by 2-dimensional thin layer chromatography (2D-TLC) using chloroform:methanol:water [65:25:4 (v/v)] for the first dimension and chloroform:methanol:acetic acid:water [90:15:10:3.5 (v/v)] for the second dimension. Lipids were visualized by cupric sulfate charring (300 mM CuSO_4_ × 5 H_2_O, 8.5% phosphoric acid, 7 min at 70°C). The TLC plates were scanned using an Epson perfection V700 Photo (Epson) scanner. Relative lipid amounts were calculated from spot densities using the AlphaEaseFC software.

### Quantification of OMV Production

The relative quantity of isolated OMVs from *A. tumefaciens* strains was determined by measuring the protein and lipid content. Protein concentration was determined by the BCA assay (Pierce, Thermo Fisher, Waltham, MA, United States) according to manufacturer’s instruction. The lipid content was measured using the lipophilic membrane dye FM4-64 (Thermo Fisher) as described in different reports (McBroom et al., 2006; Frias et al., 2010; Pérez-Cruz et al., 2016). Briefly, an aliquot of OMVs was incubated with FM4-64 (5 μg/mL in PBS) for 30 min at 30°C. OMVs alone and the FM4-64 probe alone were included as negative controls. FM4-64-labeled OMVs were washed three times and suspended in 200 μL PBS buffer. Fluorescence measurements were performed with the Infinite M Nano + multiplate reader (Tecan; λ_EX_ = 525 nm, λ_EM_ = 705 nm). Protein- and lipid-based measurements were normalized to the OD_600_ of the cell culture.

### Analysis of OMV Interaction With Bacteria

Outer membrane vesicle interaction with bacteria was assayed as described by Tashiro et al. (2017) with some modifications. Bacteria were cultivated as described above until early stationary phase, harvested, and washed three times with PBS buffer. The OD value of the respective cells was adjusted to 1.0 in 162 μL PBS buffer. Afterwards, 5 μL of FM4-64 labeled OMVs with a protein concentration of 80 μg/mL were added to the cell suspensions and samples were incubated for 30 min at 30°C. As negative control, cells incubated with 5 μL buffer were included. Cells were harvested, washed once with PBS, and resuspended in 110 μL PBS. For quantification of the OMV-bacteria cell interaction, 50 μL of the OMV-cell mixtures were transferred to 96 well plates (Corning 96 Well Half-Area Microplate flat bottom, black polystyrene, Merck, Darmstadt, Germany) and fluorescence was recorded with Infinite M Nano+ (Tecan) with the following settings: λ_EX_ = 525 nm, λ_EM_ = 705 nm, 240% gain, and *Z*-position: 20674 μm. The red fluorescence signal of bacterial cells was buffer-corrected and normalized to the initially measured OMV fluorescence.

For fluorescence-microscopy analysis 10 μL of the OMV-cell mixtures were incubated with 0.5 μg/mL Hoechst33342 (DNA stain dissolved in H_2_O, Thermo Fischer Scientific) for 5 min at RT to label viable cells.

### FM4-64 Labeling of Cells

*Agrobacterium tumefaciens* Atu8019^HIS^ and Atu8019C22A^HIS^ production strains and cells carrying the empty vector (EV) were cultivated in LB medium with 0.1 mM IPTG and respective antibiotic to stationary phase. Cells were harvested, washed twice in TBS buffer and the OD_600_ value was adjusted to 1.0. For fluorescence labeling, 100 μL of the cell suspensions were mixed with 50 μg/mL FM4-64 for 5 min at RT.

### Fluorescence Microscopy

Fluorescence microscopy and image acquisition were carried out using an Olympus BX51 microscope equipped with CCD camera (Retiga 3, QImaging), and LED light source (SOLA-365, Lumencor) driven by VisiView^®^ 3.× (Visitron systems) software. All images were acquired using a Plan-APO 100×/1.4 NA oil objective with the following filter sets: BP 450–488, FT 495 nm, and BP 512–542 (AlexaFluor488); BP 535–580, FT 595 nm, and BP 608–680 (FM4-64); BP 360–370, FT 400 nm, and BP 420–460 (Hoechst33342). Images were processed using the Image J software (Schneider et al., 2012).

### Bioorthogonal Labeling of Lipoproteins in *E. coli*

Bioorthongonal labeling of LPPs was performed according to (Rangan et al., 2010) with modifications. *E. coli* BL21 Atu8019^HIS^ and Atu8019C22A^HIS^ production strains and *E. coli* BL21 harboring the EV were cultivated in 20 mL M9 medium supplemented with 50 μg/mL kanamycin for 5–6 h at 37°C. Protein expression was induced with 0.4 mM IPTG. For metabolic labeling, 25 μM alkyne-palmitic acid in DMSO (BaseClick) was added. As negative control, cells treated with DMSO were included. After overnight incubation at 37°C, cells were harvested (10,000 × *g* for 1 min) and washed twice with PBS. Cells were dissolved in 0.1% SDS-TEA buffer [50 mM triethanolamine, 150 mM NaCl; 0.1% (w/v) SDS] supplemented with 5 mM PMSF and lysed by sonication (10 s in Sonorex Super RK1024 sonication bath, Bandelin). The lysate was incubated for 10 min on ice. Lysozyme (0.25 μg/mL) and DNaseI (0.1 μg/mL) were added and cells were further incubated for 30 min on ice. Final concentration of SDS was adjusted to 4% with 12% SDS-TEA-buffer and a second sonication for 10 s was performed. Debris was removed by centrifugation (5,000 × *g*, 5 min, and 4°C) and the protein concentration of supernatant was determined by BCA assay. The master mix for the Cu(I)-catalyzed azide-alkyne cycloaddition (CuAAC) reaction was prepared as follows. 0.1 mM Azide-Fluor 488 (Sigma Aldrich), 0.1 mM TBTA (Tris[(1-benzyl-1H-1,2,3-triazol-4-yl)methyl]amine, Sigma Aldrich) and 1 mM CuSO_4_ × 5 H_2_O were set under protection gas (Ar-flow). Then 10 mM sodium ascorbate (freshly prepared) were added. 100 μg protein (lysate) was mixed with 4% SDS-TEA buffer to a final volume of 90 μL and set under Ar-flow in a boron-silicon glass vial. Afterwards, 10 μL master mix were added and samples were incubated for 1.5 h at RT under protection gas in the dark. Proteins were precipitated using methanol/chloroform as described before (Rangan et al., 2010). The protein pellet was dissolved in 40 μL TBS by sonication for 15 min and 10 μL 5 × SDS sample buffer were added. The samples were boiled for 5 min at 97°C and for in-gel visualization of labeled LPPs, 15 μL of samples were loaded on a 16% Schägger (2006) gel. The labeled proteins were visualized using the DyLight488 (Excitation: 488 nm, Emission: 525 nm) filter set of the GelDocTM MP imaging system (BioRad, Feldenkirchen, Germany). As loading control, the SDS gel was afterwards stained with coomassie blue. To check for recombinant protein synthesis, Western blot was performed.

### Quantification of Cell Aggregation

Quantification of cell aggregation was performed as described by De Windt et al. (2006) with modifications. Briefly, round flasks containing 20 mL LB medium supplemented with 100 μg/mL streptomycin, 300 μg/mL spectinomycin, and 0.1 mM IPTG were inoculated with bacterial cells and incubated at 30°C for 28 h. At different time points, 1 mL culture was vortexed rigorously to destroy cell aggregates and the OD_600_ (OD_TOTAL_) was measured. To determine the OD of non-aggregated cells, another 1 mL of the culture was centrifuged for 2 min at 650 × *g* and the OD_600_ of the supernatant (OD_SUPERNATANT_) was determined. The aggregation index (AI) is defined as follows: AI = (OD_TOTAL_-OD_SUPERNATANT_)/(OD_TOTAL_).

### Biofilm Formation

Biofilm formation was determined using the crystal violet assay. *Agrobacterium* strains were cultured in LB medium in 24 well plates with a starting OD_600_ of 0.5. If required, medium was supplemented with the appropriate antibiotics and 0.1 mM IPTG (overexpression strains). Cultures were incubated for 24 and 72 h at 30°C. After incubation, 50 μL crystal violet (0.5%) were added and the plate was incubated for 10 min at RT with gentle rocking. The biofilm was washed three times with 1.5 mL water and dissolved in 1 mL 100% ethanol. For biofilm quantification, absorbance at 570 nm (A_570_) was determined.

### Protein Purification and Reconstitution Into Liposomes

For protein purification, *A. tumefaciens* Atu8019^HIS^-expression cultures or *E. coli* BL21 Atu2451^HIS^-expression cultures were disrupted and total membranes were extracted, as described above. The membrane pellet was solubilized with 2% (v/v) Triton X-100 in Tris buffer (50 mM Tris–HCL, 150 mM NaCl, and pH 8.0) with gentle stirring at 4°C overnight. The mixture was diluted with Tris buffer to a final Triton X-100 concentration of 0.5% (v/v) and loaded onto a Ni-IDA column (Protino, Macherey-Nagel, Düren, Germany). Unspecific proteins were removed by washing with Tris buffer containing 0.5% Triton X-100 and increasing imidazole concentrations (20, 50, and 70 mM). Finally, protein of interest was eluted with Tris buffer containing 0.5% (v/v) Triton X-100 and 300 mM imidazole. Purification success was analyzed by SDS-PAGE and Western blot analysis. Protein concentration was determined in coomassie stained SDS gels using GelDocTM MP imaging system (BioRad).

For protein reconstitution, lipid films (3.2 mg) consisting of 99.9 mol% DOPC (1,2-dioleoyl-*sn*-glycero-3-phosphatidylcholine, Avanti polar lipids), and 0.1 mol% *N*-NBD-PE [1,2-dipalmitoyl-*sn*-glycero-3-phosphoethanolamine-*N*-(7-nitro-2-1,3-benzoxadiazol-4-yl; ammonium salt), Avanti Polar Lipids] or 99.9 mol% of DOPC and 0.1 mol% DiI (1,1-Dioctadecyl-3,3,3,3-tetramethylindocarbocyanine perchlorate, AAT Bioquest) were prepared under N_2_-flow. The lipid films were rehydrated with 1 mL Tris buffer supplemented with 1% (v/v) Triton X-100 by vortexing with glass beads. Purified protein (10 μg of Atu8019 and 12 μg of Atu2451) was added followed by a 30-min incubation at RT with end-over-end rotation. Then, bio-beads SMII (100 mg; BioRad) were added and the mixture was incubated at RT for 4 h followed by addition of 200 mg SM-2 beads and incubation at 4°C for 12 h. Protein-free liposomes were prepared similarly by replacing purified protein with Tris buffer.

For density flotation, samples were mixed in an equal volume of 60% (w/v) sucrose in floating buffer in 11 × 60 mm centrifuge tubes (Beckmann) and overlaid with 1 mL each of 25 and 0% (w/v) sucrose steps in floating buffer. After centrifugation (150,000 × *g*, 6 h, and 4°C), eight fractions were collected from the top for fluorescence and protein analysis.

Successful reconstitution was confirmed by proteinase K protection assays as described above for the OMVs.

### Giant Unilamellar Vesicle (GUV) Preparation

For GUV formation, 5 μg DOPC and 0.1 mol% *N*-AlexaFluor488-PE were mixed in 10 μL chloroform:methanol [1:1 (v/v)] and 5 μL were spotted onto two platinum electrodes. After solvent evaporation under vacuum, both electrodes were assembled to one teflon chamber filled with 340 μL swelling buffer (200 mM sucrose, 2 mM Tris, 4 mM NaCl, and pH 8.0). Electroformation was performed overnight at RT using 10 Hz AC electrical field with an amplitude change of 20–1100 mV every 6 min. Subsequently, GUVs were detached from the electrodes for 30 min at 4 Hz, and 1100 mV. The GUVs were used within 24 h for interaction studies and stored at 4°C.

### Interaction of Proteoliposomes With GUVs and Bacteria

For liposome-GUV interaction studies, we used DiI-labeled liposomes. GUVs (20 μL) were incubated with DiI-labeled empty liposomes (4 μL), Atu8019^HIS^ or Atu2451^HIS^ proteoliposomes for 5 min at RT. Afterwards, 50 μL microscopy buffer (270 mM glucose, 2 mM Tris, 4 mM NaCl, and pH 8.0) were added and the mixture was spotted onto a cover slide. GUVs were let to sediment for 5 min. Samples were examined under a Leica microsystems TCS SP8 confocal laser scanning microscope using a 63× water objective. Laser lines and notch filters used during confocal microscopy: λ_EX_ = 488 nm, λ_EM_ = 525 nm, notch filter AlexaFluor488 (*N*-AlexaFluor488-PE); λ_EX_ = 550 nm, λ_EM_ = 569 nm, and notch filter TexasRed^®^ (DiI). Images were processed using the LasX (Leica microsystems) software.

Target bacteria were prepared as described for the OMV-cell interaction assay. Target bacteria and DiI-labeled proteoliposomes were mixed at RT for 2 h in TBS buffer (10 liposomes per cell). Target bacteria were stained with Hoechst33342 for 5 min at RT and liposome-cell mixtures were analyzed by fluorescence microscopy.

### Quantification of *A. tumefaciens* Attachment to *Arabidopsis thaliana* Roots

The ability of different *A. tumefaciens* derivatives to attach to *Arabidopsis thaliana* roots was analyzed according to Petrovicheva et al. (2017). *A. tumefaciens* wildtype and *atu8019* deletion strains carrying the EV and the *atu8019* overexpression strain (p8019) were grown in LB medium with 0.1 mM IPTG for 5 h at 30°C. Afterwards, cells were washed once in PBS buffer and cell concentration was adjusted to 10^8^ cells per mL. *A. thaliana* Columbia-0 seeds were cold treated as described in Wu et al. (2014) prior to seed decollation onto Murashige and Skoog plates (4.32 *g* MS salts, 0.5 *g* MES, 10 *g* sucrose, 0.8% agar in 1 L H_2_O, and pH 5.7). Seedlings were grown for 14 days at 24°C in a phytochamber (24 h/day, 10 h light, and 14 h/dark). Roots were carefully decollated and cut in 0.5 cm fragments, before 10 root segments were incubated with 10^8^ *A. tumefaciens* cells for 2 h at 30°C with end-over-end rotation. Root fragments in PBS puffer served as negative control. Unbound bacteria were removed carefully from the root segments. The DNA from root bundles with bound bacteria was extracted (mericon kit; Qiagen) to quantify the yields of attached bacteria by qPCR using primer for an *A. tumefaciens* specific gene (*chvE*). The qPCR reaction mixture contained 2 μL of the isolated DNA, 5 μL 2× iTaq universal SYBR green supermix (BioRad), 2.5 pM chvE forward and reverse primers (Supplementary Table S2) and 2.5 μL nuclease free water. The PCR reaction was run on the CFX ConnectTM real time system (BioRad) with an initial activation for 3 min at 95°C then 40 cycles of denaturation at 95°C for 10 s followed by annealing and elongation at 55°C for 30 s. To generate a standard curve for correlation of recorded Cq values to cell equivalents (CE), DNA from serial dilutions of *A. tumefaciens* wildtype cells (10^8^–10^4^) was extracted and processed through qPCR assay. The obtained regression line was y = 3.24x + 42.01 with an *R*^2^ of 0.9964 for the shown replicate. Using this standard curve, the CE values were calculated from the recorded Cq values and for representation, the Cq values in dependency of the log_10_ of CE were correlated. As internal control for DNA extraction, we added 10 ng linearized pBO6102 plasmid (carrying the FLAG-epitope) to the cell pellets prior to DNA-extraction and performed qPCRs with appropriate primer pairs specific for the FLAG-epitope (Supplementary Table S2).

### *Agrobacterium*-Mediated Transient Transformation in *Arabidopsis* Seedlings

T-DNA transfer of different *A. tumefaciens* derivatives into plant cells was analyzed by Arabidopsis seedlings infection assays using the AGROBEST method (Wu et al., 2014) as published previously (Groenewold et al., 2019). β-glucuronidase (GUS) was used as reporter to monitor T-DNA transfer.

## Results

### *Agrobacterium tumefaciens* Releases OMVs With a Specific Protein and Lipid Composition

**FIGURE 1:**
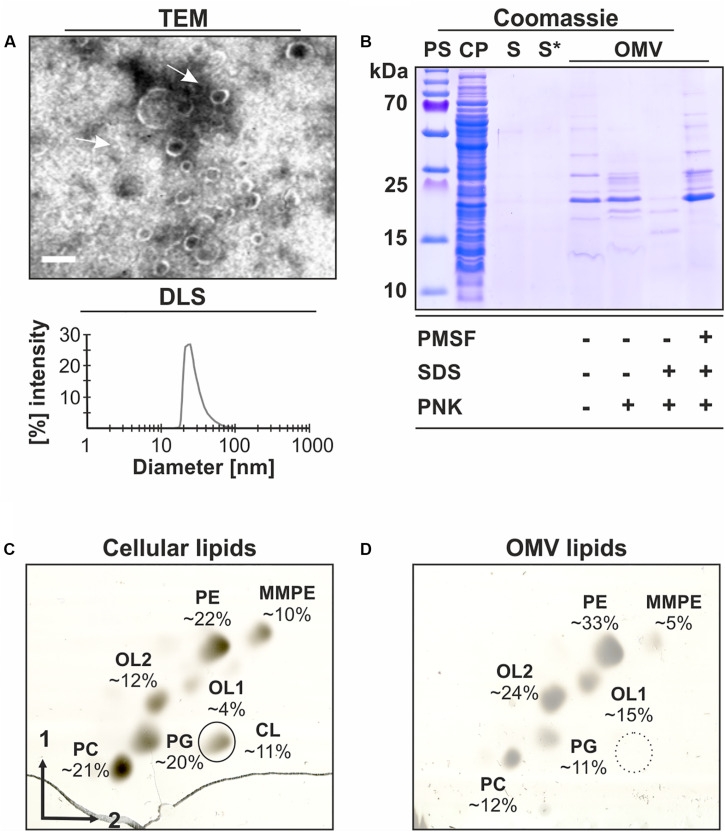
Characterization of OMVs isolated from *A. tumefaciens* culture supernatants. *A. tumefaciens* C58 cultures were grown in LB medium to an OD_600_ value of 1.0 before OMVs were isolated from the cell-free culture supernatants and further characterized. **(A)** Transmission electron microscopy (TEM) images and size distribution of OMVs determined by dynamic light scattering (DLS). The arrows indicate isolated OMVs. Scale bar: 100 nm. **(B)** SDS-PAGE analysis and proteinase K (PNK)-protection assays of OMV proteins. **(C)** Analysis of cellular and **(D)** OMV lipids by 2-dimensional TLC. Lipids were visualized and quantitated by copper (II) sulfate charring. Lipid standards were used to assign the phospholipid species. PS, protein standard; CP, cell pellet; S, cell-free culture supernatant; S*, S after ultracentrifugation containing secreted soluble proteins; OMV, pellet containing outer membrane vesicles after ultracentrifugation; CL, cardiolipin; PE, phosphatidylethanolamine; MMPE, monomethyl-PE; PC, phosphatidylcholine; PG, phosphatidylglycerol; and OL1/2, ornithine lipids 1/2.

To study OMV release and composition from *A. tumefaciens*, we isolated OMVs from cell-free supernatants of early stationary phase cultures grown in LB medium. As evident from transmission electron microscopy (TEM), the isolated OMVs were characterized by their spherical shape with a size ranging from 20–80 nm (Figure 1A, upper panel). The same vesicle size range was found by dynamic light scattering (DLS; Figure 1A, lower panel), a method estimating the hydrodynamic diameter of particles.

SDS-PAGE analysis of the OMV preparation revealed a strong reduction of the number of protein bands in comparison with whole-cell lysates (CP) and an enrichment of proteins of about 13 to 35 kDa (Figure 1B). Proteinase K treatment of the OMV fractions showed that a large number of proteins was protected by intact vesicles. This protection was abrogated, when OMV integrity was compromised by SDS. Further, the OMV proteins were protected from the proteinase in the presence of the proteinase inhibitor PMSF.

Lipids are important structural components of OMVs. To confirm the presence of vesicles in the isolated fraction and investigate the lipid composition, OMV lipids were isolated and analyzed by 2-dimensional thin layer chromatography (2D-TLC). The membrane-lipid composition of *A. tumefaciens* is well-documented and the most abundant phospholipids in this organism are phosphatidylethanolamine (PE), monomethyl-PE (MMPE), phosphatidylglycerol (PG), cardiolipin (CL), and phosphatidylcholine (PC; Wessel et al., 2006). In addition, *Agrobacterium* produces phosphate-free lipids such as ornithine lipids (OL1/2; Geske et al., 2013; Aktas et al., 2014). 2D-TLC analysis of isolated OMV lipids revealed that, except for CL, all lipids found in *Agrobacterium* cells (PE, MMPE, PC, PG, and OL1/2; Figure 1C) were also detected in the OMV fraction (Figure 1D). However, the relative proportion of the lipids differed between OMVs and cells. The presence of membrane lipids corroborated the identity of the isolated material as OMVs. Overall, our data clearly demonstrate that *A. tumefaciens* sheds OMVs into culture supernatant with a specific protein and lipid composition.

### *A. tumefaciens* OMVs Contain the Small Lipoprotein Atu8019

**FIGURE 2:**
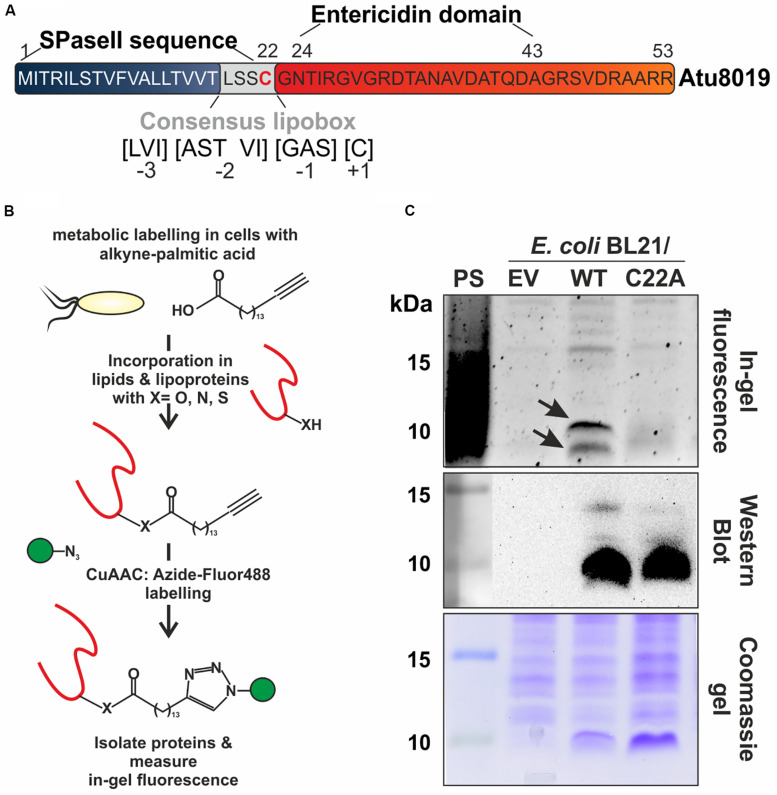
Experimental validation of Atu8019 lipidation. **(A)** Atu8019 protein sequence and predicted motifs/domains. **(B)** Schematic illustration of the lipoprotein labeling approach. **(C)** *E. coli* BL21 expression cultures containing the pET24 empty vector (EV) or producing one of the Atu8019^HIS^ derivatives (WT or C22A) were grown over night with alkyne-palmitic acid. After labeling, cells were harvested, lysed, and a copper (I)-catalyzed azide-alkyne cycloaddition (CuAAC)-reaction with Azide-Fluor488 (AF488) was performed for in-gel fluorescence profiling of lipoproteins (top). The arrows indicate acylated Atu8019 protein before and after signal-peptidase II cleavage. Atu8019^HIS^ production was verified by Western blot (middle) and the coomassie blue-stained gel served as loading control (bottom). PS, protein standard.

To identify the protein cargo of *A. tumefaciens* OMVs, we applied mass spectrometry (MS) and found 52 proteins with high confidence in at least two of three independent biological replicates (Supplementary Table S3). Among these was the small protein Atu8019 (53 aa), which is annotated as an entericidin A/B family lipoprotein. Details for MS-based identification of Atu8019 in OMVs are given in Supplementary Tables S4, S5. Entericidin proteins are proposed to be pore-forming lipopeptide toxins with bacteriolytic activity (Bishop et al., 1998). Protein analysis tools such as LipoP (Juncker et al., 2003) and PrediSi (Hiller et al., 2004) predict an N-terminal lipoprotein signal-peptidase cleavage site with a consensus lipobox, a hallmark of LPPs (Figure 2A). Like in canonical lipoboxes, it contains a conserved cysteine (C22) at position +1, which is typically acylated during the maturation process. Thus, Atu8019 very likely represents a membrane-active lipoprotein, which is post-translationally lipidated and processed. The mature protein is expected to comprise 32 aa (Figure 2A).

To validate the predicted lipid modification of Atu8019 experimentally, we performed a two-step bioorthogonal-labeling strategy using alkyne-fatty acids (Rangan et al., 2010). Briefly, after labeling *E. coli* BL21 cells producing C-terminally HIS-tagged Atu8019 from a pET24b expression vector with alkyne-palmitic acid, the LPPs in the cell lysate were labeled with Azide-Fluor488 using a Cu(I)-catalyzed azide-alkyne cycloaddition (Figure 2B). The labeled LPPs were then visualized by in-gel fluorescence (Figure 2C top). Atu8019 synthesis was verified by Western blot analysis using anti-HIS antibodies (Figure 2C, middle) and a coomassie-stained gel served as loading control (Figure 2C, bottom). As negative controls, we used strains expressing the EV or the Atu8019C22A variant, which should no longer be lipidated due to the loss of the invariable cysteine 22. Two prominent fluorescent bands with molecular masses around 10 kDa were detected in the atu8019 expressing *E. coli* strain (Figure 2C, top). These bands were missing in the control strains confirming the proposed lipidation of Atu8019 at cysteine 22. The presence of two bands is most likely due to the acylated Atu8019 protein before and after signal-peptidase II cleavage.

### Atu8019 Is an Outer Membrane Protein, Which Is Exposed at the Surface of Cells and OMVs

**FIGURE 3:**
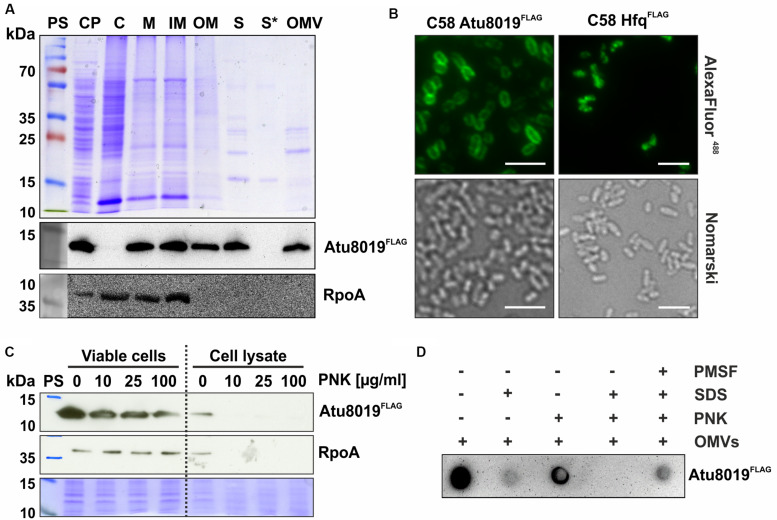
Localization of Atu8019^FLAG^ in *A. tumefaciens*. **(A)** SDS-PAGE and Western blot analysis of different cell fractions from the Atu8019^FLAG^ reporter strain. Cells were cultivated in LB medium until stationary growth phase. The cell pellet (CP) was resuspended in buffer and cells were disrupted and separated into cytosolic (C) and membrane fractions (M) by ultracentrifugation. The membrane fraction was treated with *N*-lauroylsarcosine to separate it into inner and outer membranes (IM/OM). For the extracellular localization of proteins, the cell-free culture supernatant (S) was fractionated into secreted soluble proteins (S*) and OMV-associated proteins (OMV) by ultracentrifugation. Atu8019^FLAG^ was detected with anti-FLAG antibodies and RpoA, detected by a specific antibody, served as a cytoplasmic, and inner-membrane reference protein. **(B)** Immunofluorescence imaging of Atu8019^FLAG^. Hfq^FLAG^ served as cytosolic reference protein. Scale bars: 5 μm. **(C)** Surface exposure of Atu8019^FLAG^ in cells. Proteinase K (PNK)-protection assays were performed with viable and lysed Atu8019^FLAG^ reporter strain cells to determine the orientation of Atu8019 in the outer membrane. **(D)** Surface exposure of Atu8019 in OMVs. PNK-protection assay and Dot blot with intact and SDS-treated OMVs isolated from an Atu8019^FLAG^ reporter strain.

For a detailed characterization of Atu8019 localization in cells and OMVs, we constructed an *A. tumefaciens* strain expressing a C-terminally FLAG-tagged Atu8019 derivative from the authentic locus on the chromosome. Protein localization was monitored by subcellular and extracellular fractionation. To evaluate the separation quality of the isolated subcellular and extracellular fractions, we used RpoA as marker protein for cytosolic and inner membrane fractions. As expected from previous studies (Lasserre et al., 2006; Waite et al., 2012) RpoA was detected in cytosol and inner membrane fractions but not in the outer membrane or OMVs (Figure 3A). Atu8019 was found in the inner and outer membrane (Figure 3A). Membrane localization was confirmed by immunofluorescence microscopy. In contrast to the cytosolic control protein Hfq^FLAG^, Atu8019^FLAG^ exhibited a distinct homogenous membrane location (Figure 3B). Most importantly, we also detected Atu8019 in the cell-free supernatant (S), clearly showing that the protein is secreted. Fractionation of the supernatant into secreted soluble proteins (S^∗^) and OMV-associated proteins revealed that Atu8019 is OMV associated (Figure 3A) consistent with the MS data.

Outer membrane LPPs can be either located at the inner surface of the outer membrane or exposed to the outer surface. To investigate whether Atu8019 is displayed at the surface, proteinase K (PNK) accessibility assays with cells and OMVs were performed. Atu8019 was detected by Western blot (cells) or Dot blot (OMVs) analysis using FLAG-specific antibodies. RpoA served as a proteinase-sensitive intracellular control protein. As expected, RpoA was stable in viable cells and completely degraded in lysed cells. In contrast, some Atu8019 molecules were degraded, even in intact cells, indicating that a pool of Atu8019^FLAG^ was surface exposed and accessible to proteinase digestion (Figure 3C). Similar to the situation in cells, some Atu8019 molecules were degraded in intact OMVs (Figure 3D), demonstrating that a pool of the protein is facing the vesicle surface.

### Overproduction of Atu8019 in *A. tumefaciens* Enhances Cell Aggregation and Biofilm Formation

**FIGURE 4:**
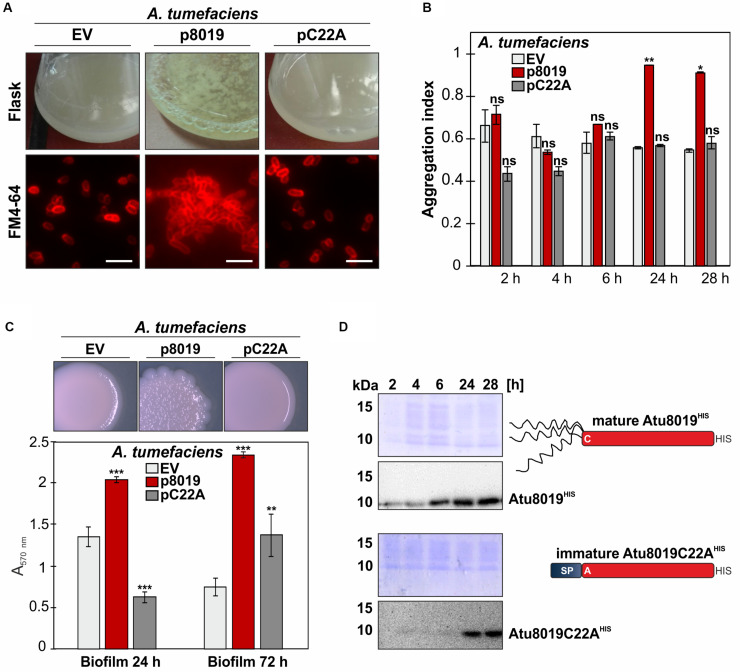
Overproduction of Atu8019 in *A. tumefaciens* enhances cell clumping and biofilm formation. **(A)** Enhancedcell aggregation by Atu8019-overproduction in*A. tumefaciens*. *Agrobacterium* strains transformed with the empty vector (pTrc200; EV), pTrc-Atu8019^HIS^ (p8019), or pTrc-Atu8019C22A^HIS^ (pC22A) were cultivated in LB medium supplemented with 0.1 mM IPTG at 30°C for 24 h. Cells were stained with the membrane dye FM4-64 and visualized by fluorescence microscopy (below). **(B)** Quantification of cell aggregation. Values are averages of triplicate assays and error bars are standard deviations. **(C)** Morphology and biofilm formation. Strains were cultivated on LB-agar plates supplemented with IPTG for 4 days before colony morphology was documented (top). Quantitative crystal violet staining biofilm assay at 24 and 72 h (bottom). Values were normalized to culture OD_600_ and are averages of quintuplicate assays. Error bars are standard deviations. **(D)** Verification of protein synthesis by Western blot. The *P*-values noted a * are less than 0.05, ** are less than 0.01, and *** are less than 0.001. Statistical testing was performed in Excel using *T*-test with unequal variance and two-tail hypothesis. ns, not significant.

To investigate the biological role of Atu8019, we constructed a markerless *atu8019* deletion and an overexpression strain and screened for phenotypic changes. For overexpression, the corresponding *atu8019* gene was cloned into the pTrc200, a broad host and IPTG-inducible expression vector to obtain protein with a C-terminal HIS-tag. Overproduction of Atu8019^HIS^ slightly delayed *E. coli* growth (Supplementary Figure S1). However, despite the predicted entericidin domain, cell lysis upon overproduction of Atu8019^HIS^ was not observed in *E. coli* or in *A. tumefaciens* (Supplementary Figures S1, S3B). Overall, *A. tumefaciens* deletion and overexpression strains showed wildtype-like phenotypes concerning growth, susceptibility against different antibiotics and ions or oxidative stress, motility, plant attachment, and plant transformation (Supplementary Figures S2, S3). Moreover, biofilm formation of the *atu8019* mutant was similar to the wildtype strain (Supplementary Figure S3D). Quantification of OMV yields by determination of their lipid and protein content suggested increased OMV formation by the *atu8019* overexpression strain (Supplementary Figure S4). The most obvious phenotype after 24 h overproduction of Atu8019 in *A. tumefaciens* was the formation of visible clumps in liquid culture (Figure 4A, above). This phenotype was also observed when we overproduced tag-less recombinant Atu8019 (Supplementary Figure S5) demonstrating that the C-terminal tag does not compromise function of the protein. The aggregation phenotype was confirmed by fluorescence microscopy of FM4-64-labeled cells (Figure 4A, below). For quantitative analysis, we determined the aggregation index (AI) after 2, 4, 6, 24, and 28 h of *atu8019* expression (Figure 4B). In the first 6 h of expression, similar AIs were determined for *Agrobacterium* expressing the empty vector (EV) or producing the Atu8019C22A derivative. A substantial increase in the AI was recorded after 24 h of wildtype-*atu8019* overexpression. Furthermore, overproduction of Atu8019 changed colony morphology and increased biofilm formation (Figure 4C). Importantly, overproduction of the immature Atu8019C22A variant in *A. tumefaciens* did not change colony morphology or induced clumping and only slightly increased biofilm formation after 72 h. Interestingly, however, biofilm formation after 24 h of the Atu8019C22A producing strain was reduced compared to the wildtype strain (Figure 4C). Localization studies of Atu8019C22A revealed that its translocation to the OM and secretion by OMVs was impaired (compare Supplementary Figures S6A,B). Thus, OM and/or OMV localization of Atu8019 seems to be crucial for the enhanced cell aggregation and biofilm phenotype. As evaluated by Western blot analysis, less amounts of C22A accumulated after 2, 4, and 6 h of expression compared to the wildtype-protein. However, after 24 h of expression similar amounts of proteins were produced of both wildtype-protein and the C22A variant (Figure 4D). All these results indicate that Atu8019 might be involved in cell-cell and/or OMV-cell adhesion.

### *Agrobacterium* OMVs Attach to Gram-Negative Bacteria

**FIGURE 5:**
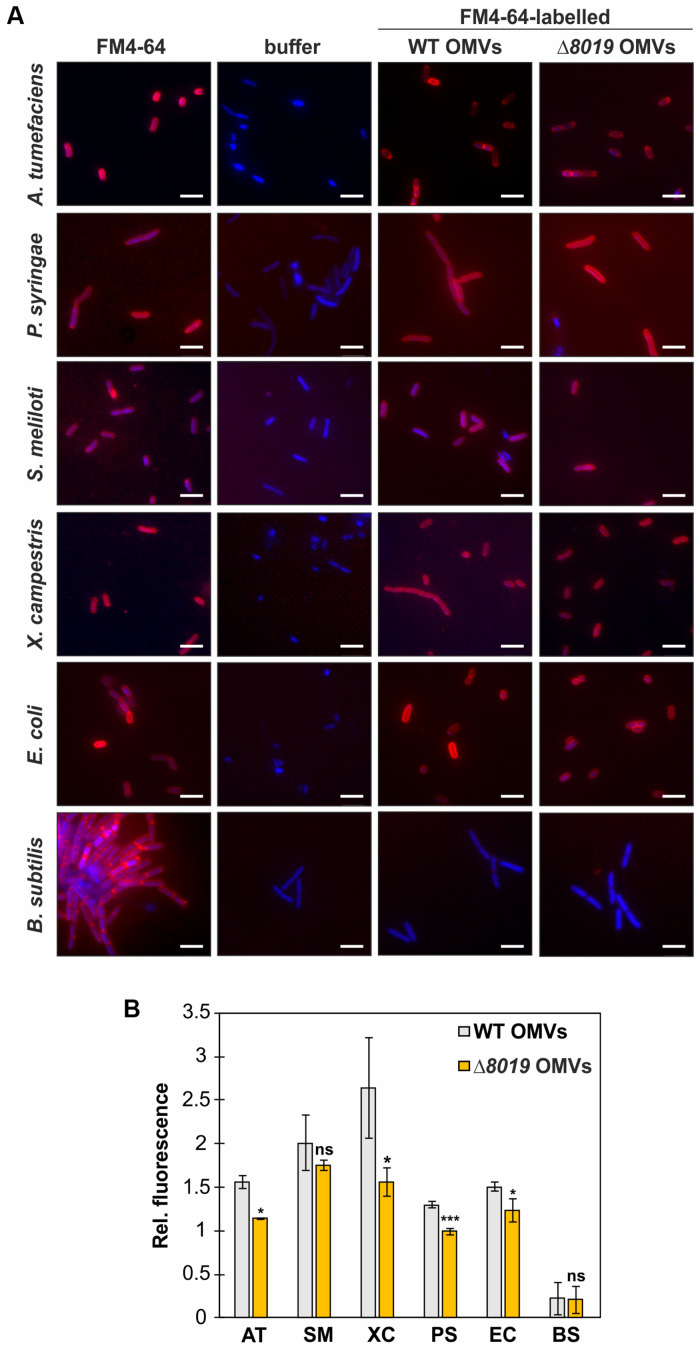
*A. tumefaciens* OMVs attach to Gram-negative bacteria. **(A)** OMV-attachment to different bacteria visualized by fluorescence microscopy. Equal amounts of FM4-64-labeled OMVs isolated from wildtype or the *atu8019*-mutant were incubated with equal amounts of Hoechst33342 (blue)-stained cells. As controls, bacteria directly stained with FM4-64, and FM4-64-free buffer controls were included. Scale bars: 5 μm. **(B)** Quantification of OMV-attachment to bacteria by determination of the relative fluorescence of cells, which were incubated with equal amounts of FM4-64 labeled OMVs. Values are averages of quadruplicate assays and error bars are standard deviations. AT, *Agrobacterium tumefaciens*; SM, *Sinorhizobium meliloti*; XC, *Xanthomonas campestris*; PS, *Pseudomonas syringae*; EC, *Escherichia coli*; and BS, *Bacillus subtilis*. The *P*-values noted a *are less than 0.05, **are less than 0.01, and ***are less than 0.001. Statistical testing was performed in Excel using *T*-test with unequal variance and two-tail hypothesis. ns, not significant.

To study whether Atu8019 is involved in OMV attachment to cells, we compared interaction of the OMVs derived from the wildtype and *atu8019* deletion strains with a range of bacteria. Prior to this analysis, the size, protein profile, and lipid composition of OMVs isolated from the Δ*atu8019* strain were characterized. Importantly, the results show that loss of *atu8019* did not affect OMV production and properties in general (Supplementary Figures S7A–D). Next, the interaction of FM4-64-labeled OMVs (red-fluorescent) from the wildtype and the *atu8019* mutant with different bacteria was visualized by fluorescence microscopy. Bacteria were labeled with Hoechst33342 (blue). In case of an attachment or fusion of the red-labeled OMVs with bacteria we expected blue cells surrounded by a red halo. As target bacteria, we used the parental strain, the Gram-negative plant-interacting *S. meliloti*, *X. campestris*, and *P. syringae* as well as *E*. *coli*, and the Gram-positive *Bacillus subtilis*. *A. tumefaciens* OMVs derived from wildtype or the *atu8019* mutant strain did not interact with *B. subtilis.* In contrast, a red-labeled surface of all other tested Gram-negative bacteria indicated an interaction (Figure 5A). These results demonstrate that *A. tumefaciens* OMVs associate with the surface of Gram-negative bacteria. At first glance, this process was independent of Atu8019 but quantification of OMV fluorescence revealed a subtle but reproducible reduction of attachment of Δ*atu8019*-derived OMVs to almost all tested Gram-negative bacteria (Figure 5B). However, the subtle reduction of attachment to *S. meliloti* did not reach statistical significance. In summary, *A. tumefaciens* OMVs have the propensity to associate with Gram-negative cells and Atu8019 is partially involved in this interaction.

### Purification and Reconstitution of Atu8019^HIS^ Into Vesicles

**FIGURE 6:**
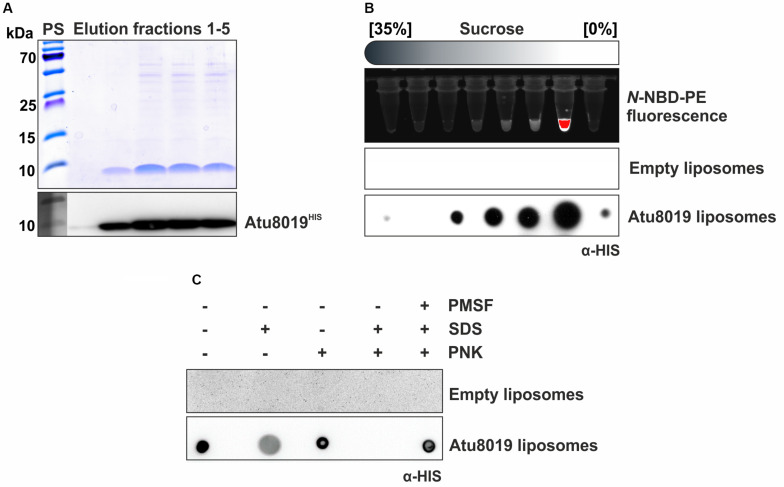
Purification and reconstitution of Atu8019^HIS^ into liposomes. **(A)** SDS gel and Western blot analysis of elution fractions after Ni-IDA purification of Atu8019^HIS^. **(B)** Efficiency of Atu8019^HIS^ reconstitution in liposomes containing the reporter lipid NBD-PE was analyzed by a flotation assay of Atu8019^HIS^ proteoliposomes in a sucrose gradient. Fractions were analyzed for NBD fluorescence (lipid marker) and protein content (Dot blot using anti-HIS antibodies). Reconstitution was evident from co-migration of phospholipid and Atu8019^HIS^. **(C)** Proteinase K (PNK)-protection assay with reconstituted liposomes. A Dot blot using anti-HIS antibodies was conducted to detect Atu8019^HIS^ in intact or SDS-treated liposomes.

To assess whether Atu8019 is involved in OMV attachment to bacteria, we purified HIS-tagged protein from Triton X-100 solubilized membranes of the *Agrobacterium atu8019* overexpression strain introduced above (Figure 6A). Purified Atu8019^HIS^ was reconstituted into liposomes containing fluorescent *N*-NBD-PE for detection. Empty liposomes served as control. Efficient reconstitution was confirmed by flotation experiments (Figure 6B). Topology of Atu8019^HIS^ in the proteoliposomes was evaluated by proteinase K digestion of intact and disrupted vesicles. Approximately 50% of the total protein was protected in intact liposomes showing that the protein is reconstituted (Figure 6C).

### Atu8019 Proteoliposomes Interact With Model Membranes

To investigate the interaction of Atu8019 proteoliposomes with model membranes, we prepared GUVs clomposed of DOPC with trace amounts of green-fluorescent AlexaFluor488-PE (0.1 mol%). The Atu8019 proteoliposomes were labeled with the red-fluorescent probe DiI. As negative controls, DiI-labeled liposomes without protein (empty liposomes) or containing the predicted lipoprotein Atu2451 were included. Atu2451 is annotated as a lysozyme-like protein and was also identified in *A. tumefaciens* OMVs by mass spectrometry (Supplementary Tables S3, S4). A HIS-tagged version of Atu2451 was overproduced from a pET24b expression vector and OMV localization was confirmed in *E. coli* (Supplementary Figure S8A). As described for Atu8019^HIS^, Atu2451^HIS^ was purified and reconstituted into DiI-labeled DOPC liposomes (Supplementary Figures S8B–D).

**FIGURE 7:**
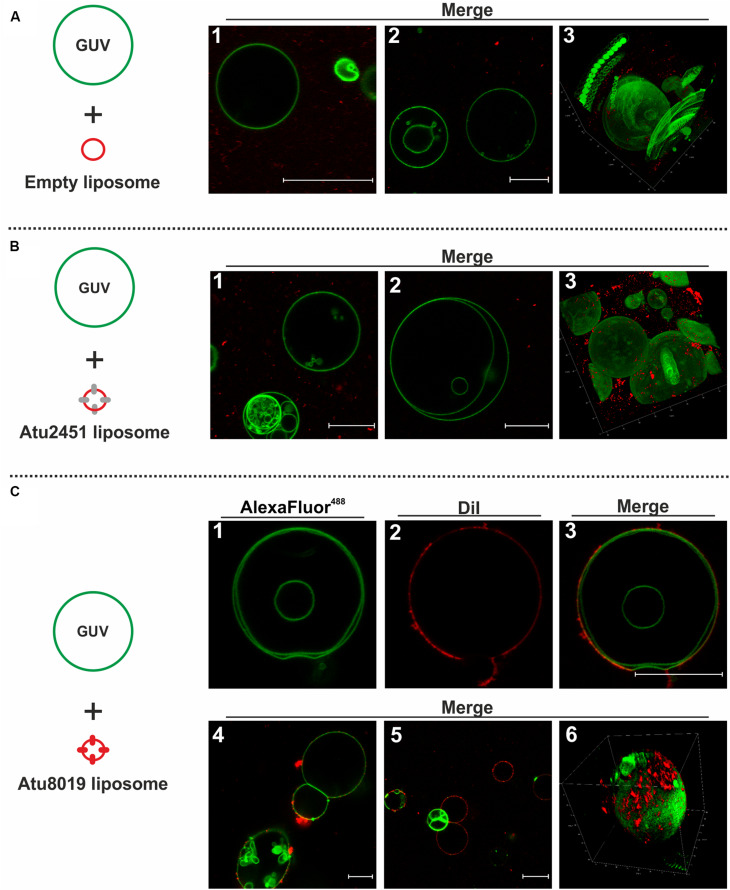
Atu8019^HIS^ proteoliposomes interact with model membranes. Interaction of Atu8019 proteoliposomes with giant unilamellar vesicles (GUVs). Left side shows a schematic illustration of the used liposomes in the corresponding experiment. Green-fluorescent GUVs (DOPC, AlexaFluor488-PE) were incubated for 5 min with red-fluorescent (proteo)liposomes (DOPC, DiI) and subjected to confocal scanning microscopy. Interaction of empty liposomes **(A)**, Atu2451 proteoliposomes (25 proteins/vesicle; **B**) and Atu8019 proteoliposomes (**C1**-**3**: 25 proteins/vesicle; **C4-6**: 100 proteins/vesicle) with GUVs. **A3**, **B3,** and **C6** represent Z-stack images. All images were obtained at RT in microscopy buffer. Scale bars: 20 μm.

DiI-labeled empty or proteoliposomes were incubated for 5 min with green-fluorescent GUVs and potential interactions were visualized by confocal scanning microscopy (Figure 7). In case of an interaction of proteoliposomes with GUVs, we expected a co-localization of the green GUVs and the red proteoliposomes. Neither empty liposomes, nor Atu2451^HIS^ proteoliposomes associated with GUVs (Figures 7A,B). In contrast, a clear association between Atu8019 liposomes and GUVs was visible as indicated by the co-localization of red-labelled Atu8019 liposomes and green GUVs (Figures 3–6, 7C). Atu8019 liposomes associated with the surface of GUVs without entering the lumen. This was further confirmed by 3-dimensional imaging (Figures 6, 7C) of the GUVs illustrating that Atu8019 proteoliposomes were attached to the GUV surface.

To examine a possible lytic activity of Atu8019, as based on its predicted entericidin domain, we performed liposome disruption assays (Ambroggio et al., 2005) using DOPC-liposomes with incorporated fluorescent probe (fluorescein) in the absence and presence of Atu8019 proteoliposomes. Membrane disruptive or pore-forming properties for Atu8019 were not observed within 15 min incubation at RT (Supplementary Figure S9).

### Atu8019 Proteoliposomes Attach to the Cell Surface of Specific Gram-Negative Bacteria

**FIGURE 8:**
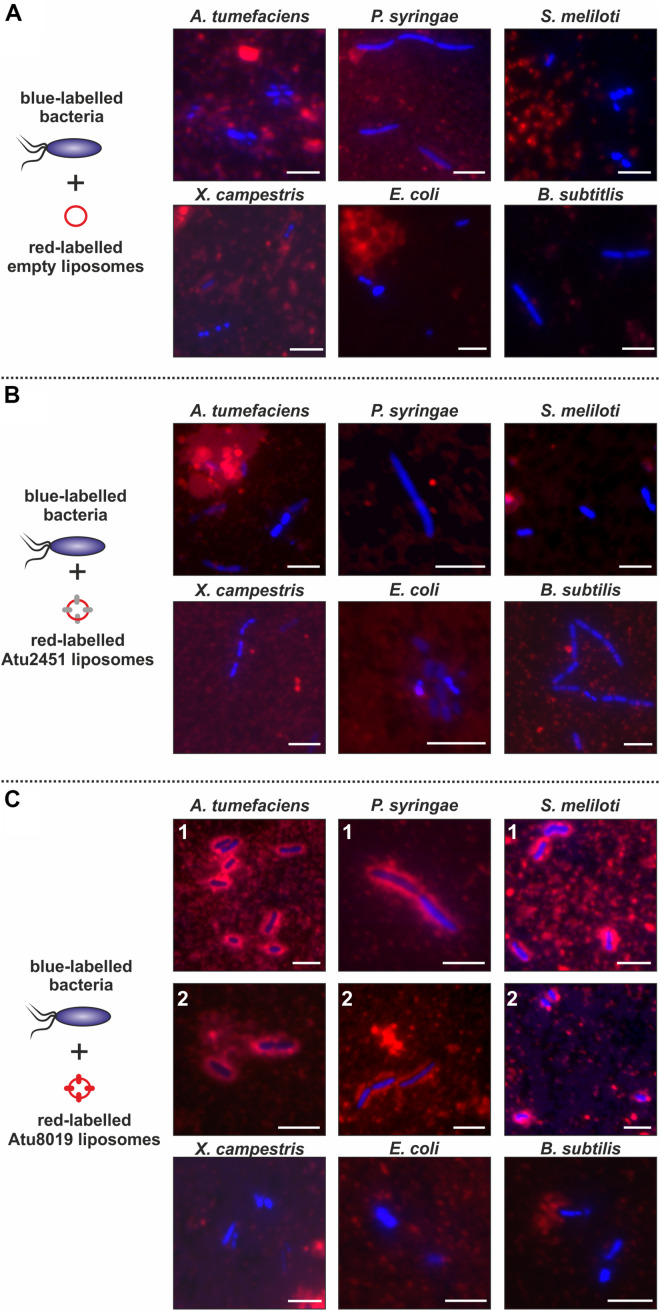
Specific interaction of Atu8019^HIS^ proteoliposomes with *P. syringae* and *S. meliloti*. Interaction of empty liposomes **(A)**, Atu2451 proteoliposomes **(B)** and Atu8019 proteoliposomes **(C)** with target cells. DiI-labeled (proteo)liposomes and Hoechst33342-labeled target bacteria were incubated for 2 h before imaging by fluorescence microscopy. Scale bars: 5 μm.

Our *in vitro* membrane-interaction studies showed that Atu8019 interacts with model membranes. Next, we investigated the interaction of Atu8019^HIS^ proteoliposomes with bacterial cells. To this end, various bacteria (introduced in Figure 5) were labeled with Hoechst33342 (blue cells) and incubated with DiI-labeled (red) Atu8019^HIS^ proteoliposomes for 2 h. An interaction between proteoliposomes and cells was monitored by fluorescence microscopy. Empty liposomes or Atu2451^HIS^ proteoliposomes did not associate with the tested strains (Figures 8A,B). Similarly, an interaction of Atu8019 liposomes with *X. campestris, B. subtilis*, and *E. coli* was not detectable (Figure 8C below). Interestingly, however, Atu8019 liposomes decorated the parental *A. tumefaciens* strain and the two plant-interacting Gram-negative bacteria *P. syringae* and *S. meliloti* (Figure 8C above). The interaction of Atu8019 with *P. syringae* and *S. meliloti* did not lyse the target cells as evident from the microscopic analyses (Figure 8C) and demonstrated by survival tests in the presence and absence of native Atu8019-enriched OMVs derived from *Agrobacterium atu8019* overexpression strain (Supplementary Figure S10). These data show that Atu8019 not only interacts with model membranes but also with some bacteria without lysing them.

## Discussion

Most bacteria release MVs with a specific cargo destined for intra- and interspecies communication. While there has been a substantial and increasing number of proteomic studies on the cargo of bacterial MVs, mechanistic details of OMV biogenesis and function are largely unknown. In this study, we investigated OMV formation and the role of the OMV-associated protein Atu8019 in the phytopathogen *A. tumefaciens*. Atu8019 is a small surface-exposed lipoprotein (mature protein: 32 aa), which is constitutively secreted by OMVs. The protein is annotated as an entericidin B homolog (EcnB). Entericidins are proposed to be pore-forming lipopeptide toxins with bacteriolytic activity (Bishop et al., 1998; Schubiger et al., 2015). EcnB homologs have been annotated in genomes of diverse species of Proteobacteria (Schubiger et al., 2015) suggesting a highly conserved but largely unexplored function.

Like in many human pathogenic bacteria, the *ecnB* gene in *E. coli* is located within the *ecnAB* operon. The *E. coli ecnAB* genes are proposed to code for a toxin-antitoxin system regulating bacteriolysis during stationary phase. Overproduction of the lipoprotein EcnB resulted in increased bacteriolysis, whereas expression of *ecnA* and *ecnB* in cis counteracted bacteriolytic activity. Hence, EcnA was designated the antitoxin neutralizing the toxin EcnB in *E. coli* (Bishop et al., 1998). If this were true, deletion of the *ecnA* gene should be lethal, which does not seem to be the case as an *ecnA* mutant is available in the *E. coli* Keio collection (Baba et al., 2006). This apparent controversy warrants further investigation of EcnB homologs.

The bacteriolytic activity of a 3 kDa secreted EcnB homolog from *Enterobacter* sp. strain C6-6 against *Flavobacterium psychrophilum* supports the toxin activity of bacterial EcnB homologs (Schubiger et al., 2015). The EcnAB toxin-antitoxin system is not restricted to human pathogens as thought previously, but is also present in *Xanthomonas* species. In the phytopathogen *Xanthomonas citri*, expression of *ecnAB* is positively regulated by quorum sensing by the RpfF system. A *X. citri ecnA*-mutant is characterized by less biofilm and extracellular polymeric substances formation, reduced sliding motility, virulence, and survival under stress conditions (Granato et al., 2019). *ecnAB* mutants of the human pathogen *Moraxella catarrhalis* exhibit a subtle decrease in adherence to the respiratory tract (de Vries et al., 2013) which is reminiscent of the attachment activity we observed with Atu8019. Interestingly, *A. tumefaciens* and many other Proteobacteria encoding an *ecnB*-like gene lack an *ecnA* homolog suggesting different protective immunity mechanism(s) or divergent functions for EcnB-like proteins in *ecnA*-lacking organisms. Indeed, the EcnB homolog Atu8019 from *A. tumefaciens* did not exhibit an obvious bacteriolytic or inhibitory effect on bacterial cells, even when bacteria were treated with Atu8019-enriched OMVs or proteoliposomes suggesting a function that differs from the proposed bacteriolytic activity.

Entericidin B homologs were detected in OMVs from different Gram-negative pathogens such as *Klebsiella*, *Shigella*, *Acinetobacter*, and different *E. coli* strains (Lee et al., 2007; Lee et al., 2012; Mendez et al., 2012; Kunsmann et al., 2015) indicating a conserved OMV-related function. To the best of our knowledge, this is the first biochemical and functional study of an EcnB-like lipoprotein with respect to its OMV-associated function. Our localization studies show that Atu8019 is a surface-exposed lipoprotein. Surface-exposed LPPs play important roles as structural proteins, antigens, toxins, nutrient-binding proteins, or adhesins in many pathogenic bacteria (Wilson and Bernstein, 2016). RNA-seq analysis shows that expression of *atu8019* is downregulated in the absence of the transcription regulator LsrB (Tang et al., 2018). The *lsrB* mutant shows several defects like reduced succinoglycan production, resistance to oxidative stress, plant attachment and transformation. This is probably due to the mis-regulation of hundreds of genes because *atu8019* was dispensable for plant-cell attachment and transformation, biofilm and OMV formation, motility, and resistance against different cations or antibiotics and oxidative stress. While deletion of *atu8019* was asymptomatic, overexpression of the gene triggered cell aggregation and biofilm formation. Moreover, the atu8019 overexpression strain produced more OMVs, which were enriched with Atu8019 protein. This prompted us to speculate that Atu8019 might mediate cell-cell or OMV-cell attachment resulting in cell clumping. In line with this notion, OMVs isolated from *Agrobacterium* adhered to various Gram-negative bacteria. OMVs isolated from the *atu8019* mutant exhibited a subtle but consistent decrease in OMV-cell attachment to all tested Gram-negative cells. Most likely, OMV attachment depends on multiple factors and not on Atu8019 alone, but our results support an involvement of Atu8019 in this process. Specific molecular patterns seem to be required since *A. tumefaciens* OMVs did not interact with the Gram-positive *B. subtilis*. There are two possible explanations for this finding. (i) LPS or OM proteins only found in Gram-negative bacteria are required for OMV-cell contact, or (ii) *A. tumefaciens* OMV-cell interaction is mediated by membrane phospholipids, which would be shielded by the peptidoglycan layer of Gram-positive bacteria. *P. aeruginosa* OMVs can attach to both Gram-negative and Gram-positive bacteria probably by salt bridging through cations. Once attached to the cell surface, *Pseudomonas* OMVs fuse with the OM of Gram-negative bacteria and release their bacteriolytic content (Kadurugamuwa and Beveridge, 1996; Tashiro et al., 2010; Tashiro et al., 2017). In contrast, attachment to Gram-positive bacteria induces liberation of autolysin from OMVs, which break down the cell wall (Kadurugamuwa and Beveridge, 1996). In case of *Agrobacterium* OMVs based on the tested strains, we have no evidence for a lytic activity. Furthermore, we cannot discriminate whether *A. tumefaciens* OMVs attach to the cell surface or fuse with the OM of the investigated bacteria. Since *Agrobacterium* OMVs adhere to all tested Gram-negative bacteria, this may be a general mechanism to exchange non-specialized cargo between bacterial species as suggested for MV-mediated DNA-transfer by Tran and Boedicker (2017). On the other hand, it is conceivable that *Agrobacterium* OMVs generally adhere to Gram-negative bacteria, and once attached to the cell surface, a species-specific cargo delivery mechanism is activated. In this context, it is interesting to note that in contrast to native OMVs, artificial Atu8019 liposomes exhibited a species-specific interaction. The Atu8019 liposomes adhered only to *Agrobacterium* cells and the two plant-interacting bacteria *S. meliloti* and *P. syringae*. These data suggest that Atu8019 confers vesicle-attachment to selected Gram-negative bacteria. The molecular mechanism(s) underlying this selective interaction and the consequences on the target strains remain(s) to be investigated. Since Atu8019 liposomes also attached to the surface of *atu8019* mutant strains (Supplementary Figure S11), we can exclude that surface-displayed Atu8019 in recipient cells is required for this vesicle-cell interaction.

The selective delivery of signals in natural mixed microbial communities is important for the survival of bacteria. Several studies indicate that surface-exposed proteins mediate selective OMV-cell interactions. MVs from *B. agrestis* are exclusively delivered to the same genus. This specific interaction is proposed to be mediated by a combination of certain physiochemical surface interactions and surface-exposed proteins (Tashiro et al., 2017). Gujrati et al. (2014) have engineered MVs, which can be trafficked to certain types of cells by changing the composition of surface proteins.

Taken together, our data revealed that *A. tumefaciens* produces OMVs with a specific cargo and lipid composition. Isolated OMVs attach to the cell surface of Gram-negative bacteria without lysing them and the small lipoprotein Atu8019, which is exposed at the OMV surface, contributes to this interaction. Interestingly, artificial liposomes containing Atu8019 do not attach to all tested but to selected Gram-negative bacteria and the parental strain suggesting a role for Atu8019 in target-specific OMV-cargo delivery. The current work lays the foundation for studying *A. tumefaciens* OMV-interactions within bacterial communities.

## Data Availability Statement

All datasets presented in this study are included in the article/Supplementary Material.

## Author Contributions

MA supervised the work and wrote the manuscript. MA, TG, and LK conceived and designed the experiments. LK has planned and performed most of the experiments and contributed in writing. JB and SS contributed to MS analyses. MA, LK, and TG carried out the data interpretation. SH and BJ helped LK in GUV and proteoliposome experiments. CF and KG assisted in cloning experiments. All authors read and approved the final manuscript.

## Conflict of Interest

The authors declare that the research was conducted in the absence of any commercial or financial relationships that could be construed as a potential conflict of interest.
