# Supplementary material for: Agrobacterium tumefaciens Small Lipoprotein Atu8019 Is Involved in Selective Outer Membrane Vesicle (OMV) Docking to Bacterial Cells
Source: Front Microbiol. 2020 Jun 9;11:1228. doi: 10.3389/fmicb.2020.01228 (PMC7296081; doi:10.3389/fmicb.2020.01228)
Supplement: Supplementary file 1 [file Data_Sheet_1.pdf]

Supplementary Material

Supplementary Figures

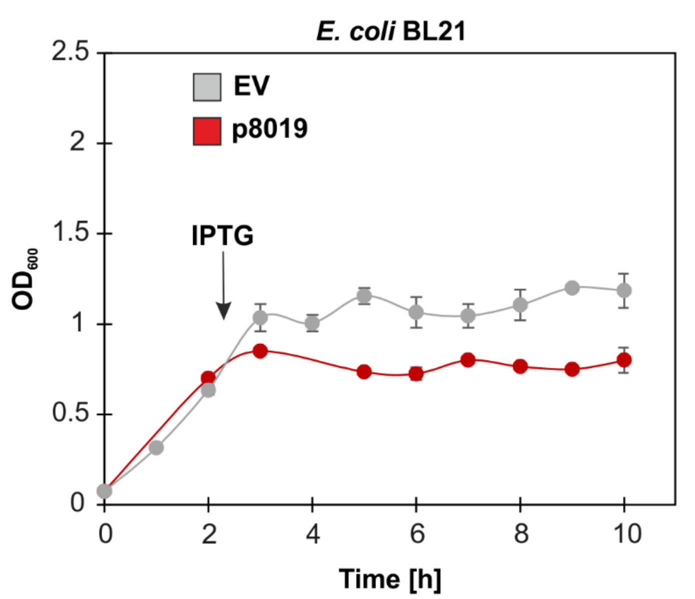

Supplementary Figure 1. Overproduction of Atu8019^{HIS} in *E. coli* does not cause cell lysis. Growth analysis of Atu8019^{HIS} overproduction strain (p8019) in LB medium at 30°C. Protein expression was induced after cultivation at 37°C at an OD_{600} of 0.6 by addition of 0.4 mM IPTG. *E. coli* cells carrying the empty vector (EV, pET24b) served as negative control.

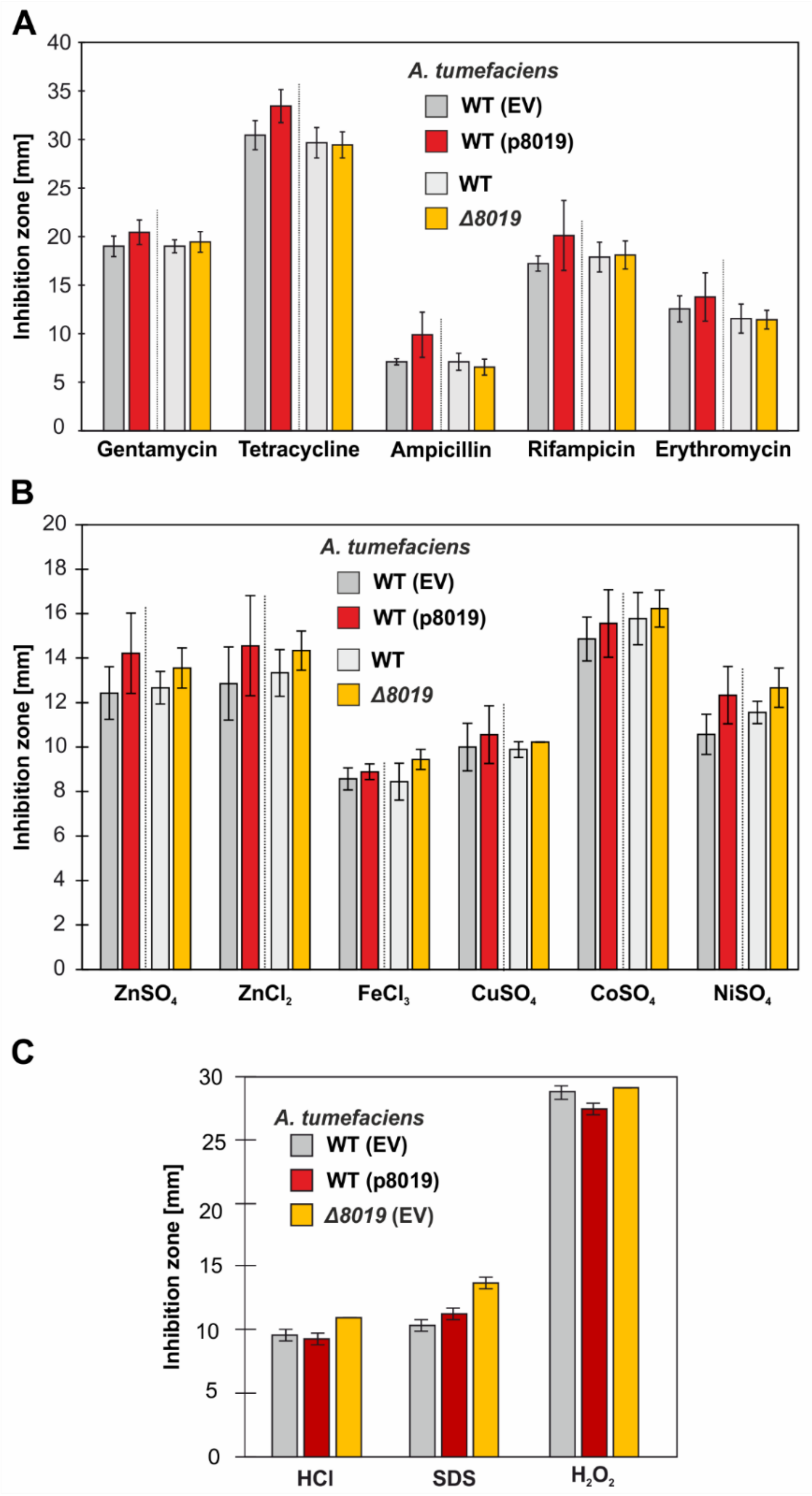

Supplementary Figure 2. Deletion or overexpression of *atu8019* does not influence stress tolerance in *A. tumefaciens*. Tolerance to different stressors of *atu8019* deletion (*Δ8019*) and *atu8019* overexpression strains (p8019) determined by agar diffusion assays. Overexpression strain and empty vector containing strains (EV; pTrc200) were cultivated in LB medium with appropriate antibiotics to an OD_{600} of 0.5 before expression was induced (0.1 mM IPTG) and the cultures were further incubated for 3 h at 30°C. *A. tumefaciens* WT and the *atu8019* deletion strain were cultivated for 6 h at 30°C in LB medium. Equal amounts of cells were plated on LB-agar plates (containing 0.1 mM IPTG and appropriate antibiotics for p8019 and EV strains) before filter discs were placed on the plates. We used 1 µL of antibiotic stock solutions (gentamycin [30 µg/mL], tetracycline [30 µg/mL], ampicillin [25 µg/mL], rifampicin [5 µg/mL], erythromycin [5 µg/mL]) and 3 µL of 1M ion stock solutions (1M), HCl (2 M), SDS (10%) and H_2O_2 (5.5 M). Inhibition zones were determined after 2 days of cultivation at 30°C. Values are averages of triplicate assays and error bars are standard deviations

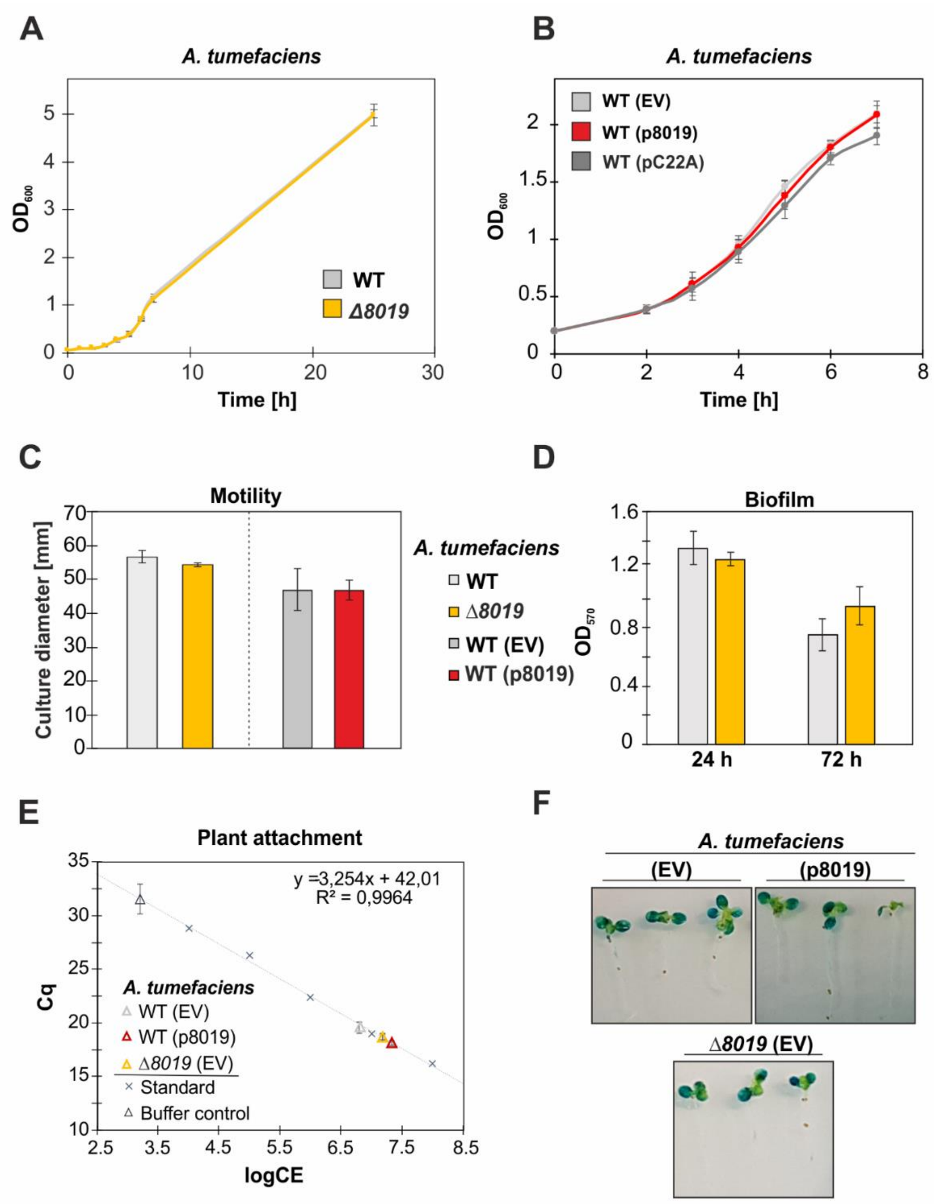

Supplementary Figure 3. Deletion of *atu8019* has no impact on motility, biofilm formation, plant attachment and transformation capacity of *A. tumefaciens*. (A) and **(B)** Growth of different *A. tumefaciens* strains in LB medium at 30°C. Overexpression strains (p8019 and pC22A) and empty vector containing strains (EV; pTrc200) were cultivated in LB medium with appropriate antibiotics and 0.1 mM IPTG. **(C)** Motility of *A. tumefaciens atu8019* deletion and overexpression strains on M9 soft agar plates with 0.25% (w/v) agar. Cultures were grown in LB medium (with 0.1 mM IPTG and appropriate antibiotic for p8019 and EV strains) at 30°C for 6 h. Cells were washed and 10 µl were spotted on soft agar plates and incubated for 72 h at 30°C before motility diameter was measured. Values are averages of triplicates and error bars are standard deviations. **(D)** Determination of biofilm formation of *A. tumefaciens* WT and Δ*8019* strain by crystal violet assay. Values are averages of quintuplicate assays and error bars are standard deviations. **(E)** qPCR-based quantification of *A. tumefaciens* attachment to *Arabidopsis thaliana* roots. *A. tumefaciens* WT and Δ*8019* strains carrying the empty vector (EV) or the pTrc-*atu8019*-expression plasmid (p8019) were grown in LB medium supplemented with 0.1 mM IPTG for 5 at 30°C. Cells (10^8) were incubated with 10 root fragments of *A. thaliana* Columbia-0 and root-bound bacteria were quantified by qPCR as described in Material and Methods. Results are presented as Cq-values compared to $\log_{10}$ of cell equivalents (CE). Values are averages of triplicates and error bars are standard deviations. **(F)** *Agrobacterium*-mediated transient transformation of *Arabidopsis* seedlings. DNA transfer capability of different *A. tumefaciens* strains into *A. thaliana* was analyzed as described by Wu et al. (Wu *et al.*, 2014). Briefly, 7-day old *A. thaliana efr*-1 seedlings were incubated with *A. tumefaciens* WT and *atu8019* deletion strain carrying the empty vector (EV) or *atu8019*-overexpression (p8019) plasmid for three days. All strains carried the pBISN1 plasmid, encoding for the *gusA* intron. Afterwards, GUS activity was analyzed by GUS staining. One representative result out of three independent triplicates is shown for each strain.

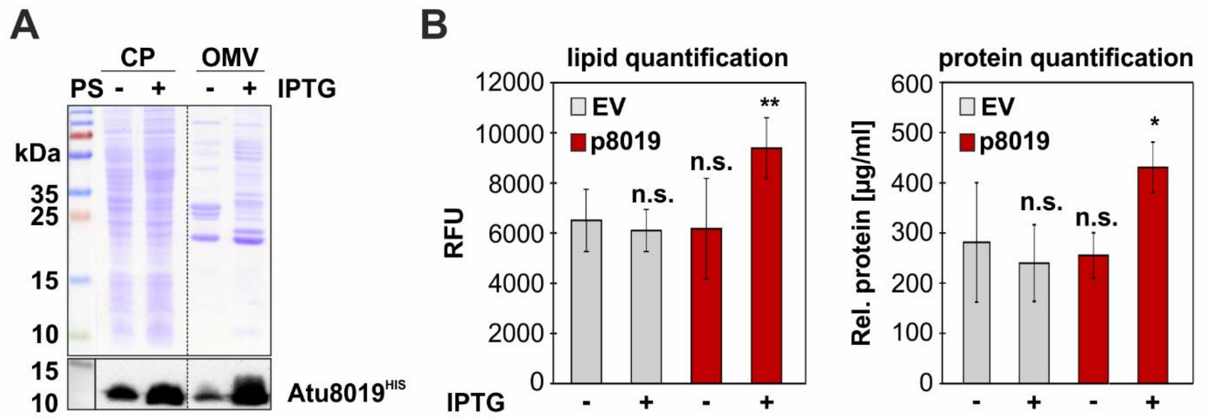

Supplementary Figure 4. Overproduction of Atu8019 in *A. tumefaciens* increases OMV formation. (A) Enrichment of Atu8019^{HIS} in *A. tumefaciens* OMVs by plasmid-based overproduction. *A. tumefaciens* WT carrying the empty vector (EV) or the *atu8019*-overexpression plasmid (p8019) were cultivated in LB medium for 24 h at 30°C with or without IPTG induction. Proteins from cell pellet (CP) and isolated OMVs were analyzed by SDS-PAGE and Western blot. Atu8019^{HIS} was detected using anti-HIS antibodies. **(B)** Influence of Atu8019 overproduction on OMV formation. OMV yields were quantified by determination of their lipid content by FM4-64 labelling. Fluorescence was recorded at 705 nm following excitation at 525 nm. The absolute fluorescence was normalized to the OD_{600} of the respective culture, to obtain relative fluorescence units (RFU). As a second approach to quantify OMV yields, concentration of OMV-proteins was determined using BCA assay and protein-concentration was normalized to OD_{600} of the OMV-producing culture. Values are averages of triplicates and error bars are standard deviations. PS, protein standard. The P-values noted a * are less than 0.05, ** are less than 0.01 and *** are less than 0.001. Statistical testing was performed in Excel using T-test with unequal variance and two-tail hypothesis. n.s., not significant.

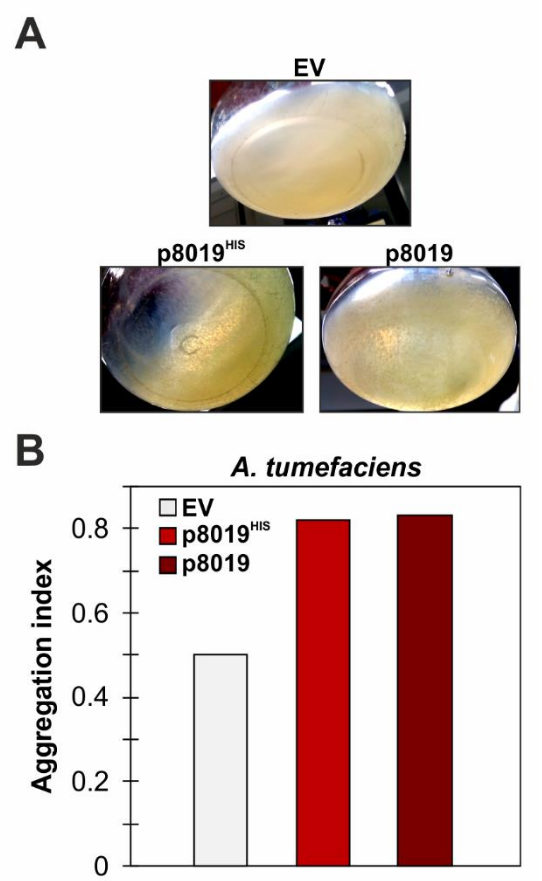

Supplementary Figure 5. The C-terminal HIS-tag does not compromise Atu8019 function. (A) Visible cell clumps of *atu8019* overexpression strains in liquid culture. Recombinant Atu8019 was overproduced as a C-terminal HIS-tagged und tag-less version in *A. tumefaciens* WT and the Aggregation index **(B)** was determined as described in Fig. 4. WT strain containing the empty vector (EV, pTrc200) served as control.

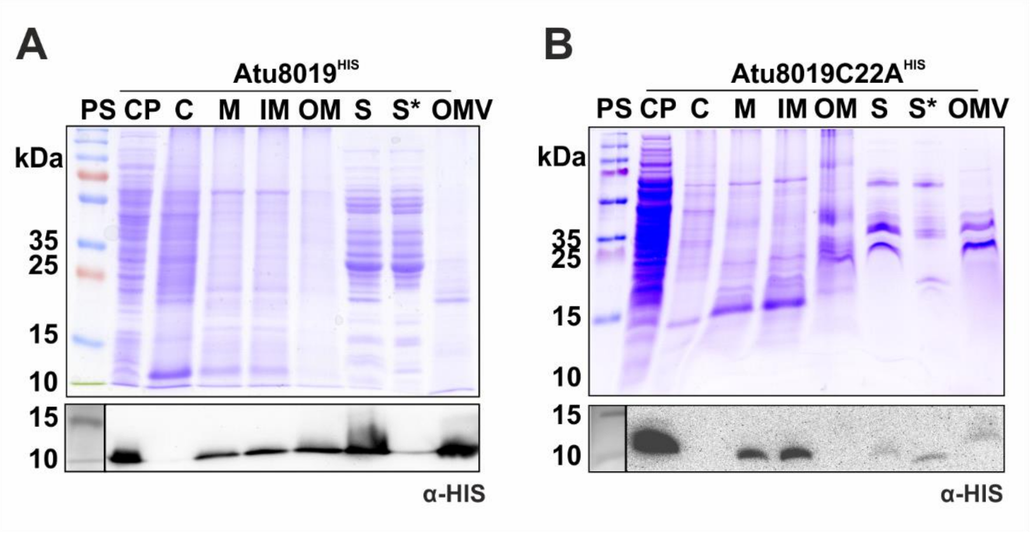

Supplementary Figure 6. The Cys22 residue is required for Atu8019 exposure and packing into OMVs. SDS-PAGE and Western blot analysis of different cell fractions from *A. tumefaciens* strains producing Atu8019^{HIS} **(A)** and Atu8019C22A^{HIS} **(B)**. Cells were cultivated in LB medium to an OD_{600} of 0.6 and induced with 0.4 mM IPTG overnight. Cells were harvested, disrupted and separated into cytosolic (C) and membrane fraction (M) by ultracentrifugation. The membrane fraction was treated with *N*-lauroylsarcosine for separation into inner and outer membranes (IM/OM). For the extracellular localization of the proteins, the cell-free culture supernatant (S) was fractionated into secreted soluble proteins (S*) and OMV-associated proteins (OMV) by ultracentrifugation. Atu8019 derivatives were detected with anti-HIS antibodies. CP, cell pellet; PS, protein standard.

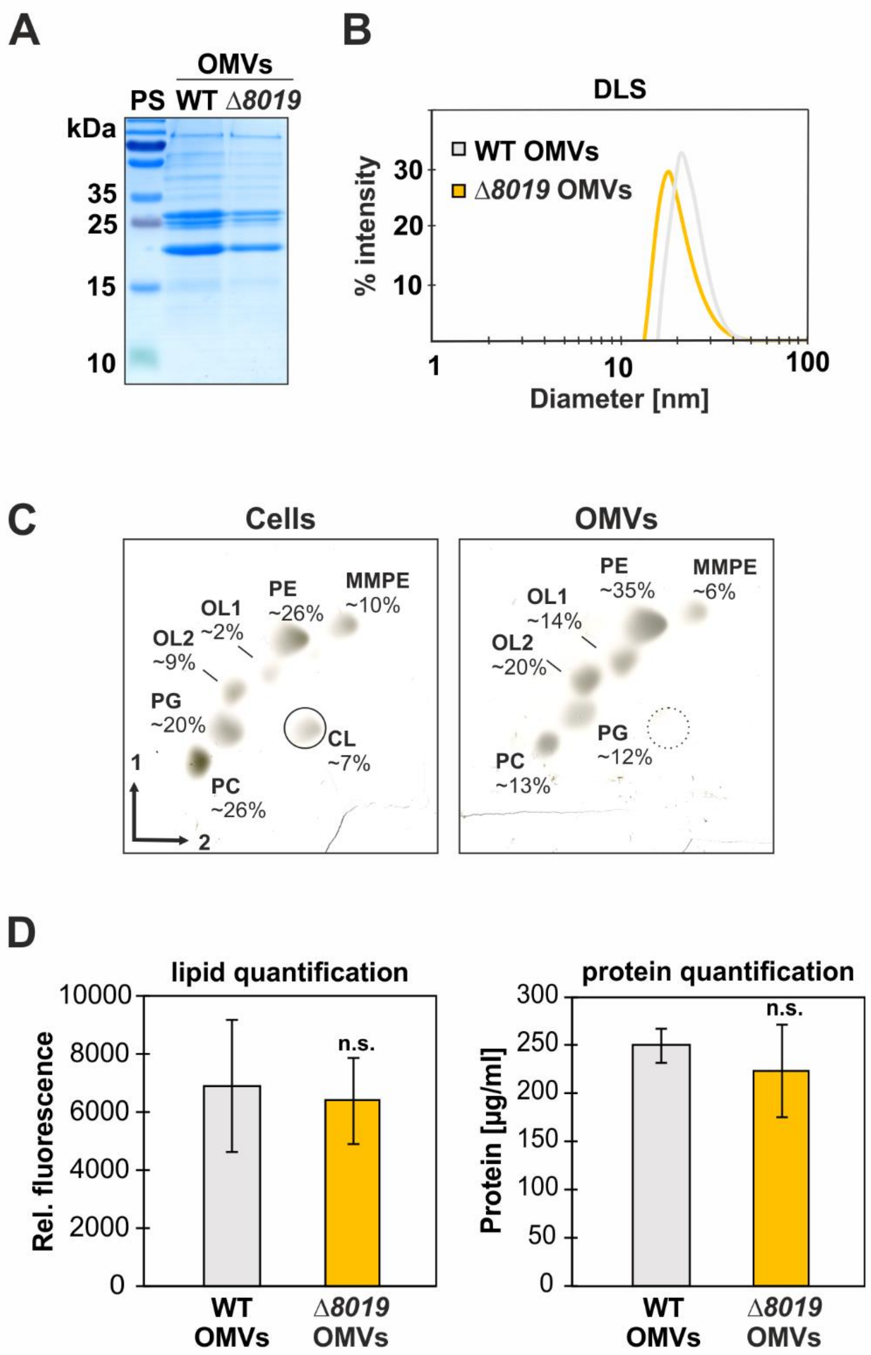

Supplementary Figure 7. Deletion of *atu8019* does not influence OMV production and properties. (A) Comparative SDS-PAGE analysis of OMV proteins secreted by *A. tumefaciens* WT and *Δ8019* strain. **(B)** Size distribution of OMVs determined by dynamic light scattering. **(C)** Analysis of cellular and OMV lipids by 2-dimensional TLC. Lipids were visualized and quantitated by copper (II) sulfate charring. **(D)** Quantification of OMV production by determination of lipid and protein concentration as described in Material and Methods. Values are averages from three independent biological replicates. Error bars represent standard deviations. CL, cardiolipin; PE, phosphatidylethanolamine; MMPE, monomethyl-PE; PC, phosphatidylcholine; PG, phosphatidylglycerol; OL1/2, ornithine lipids 1/2. Statistical testing was performed in Excel using T-test with unequal variance and two-tail hypothesis. n.s., not significant.

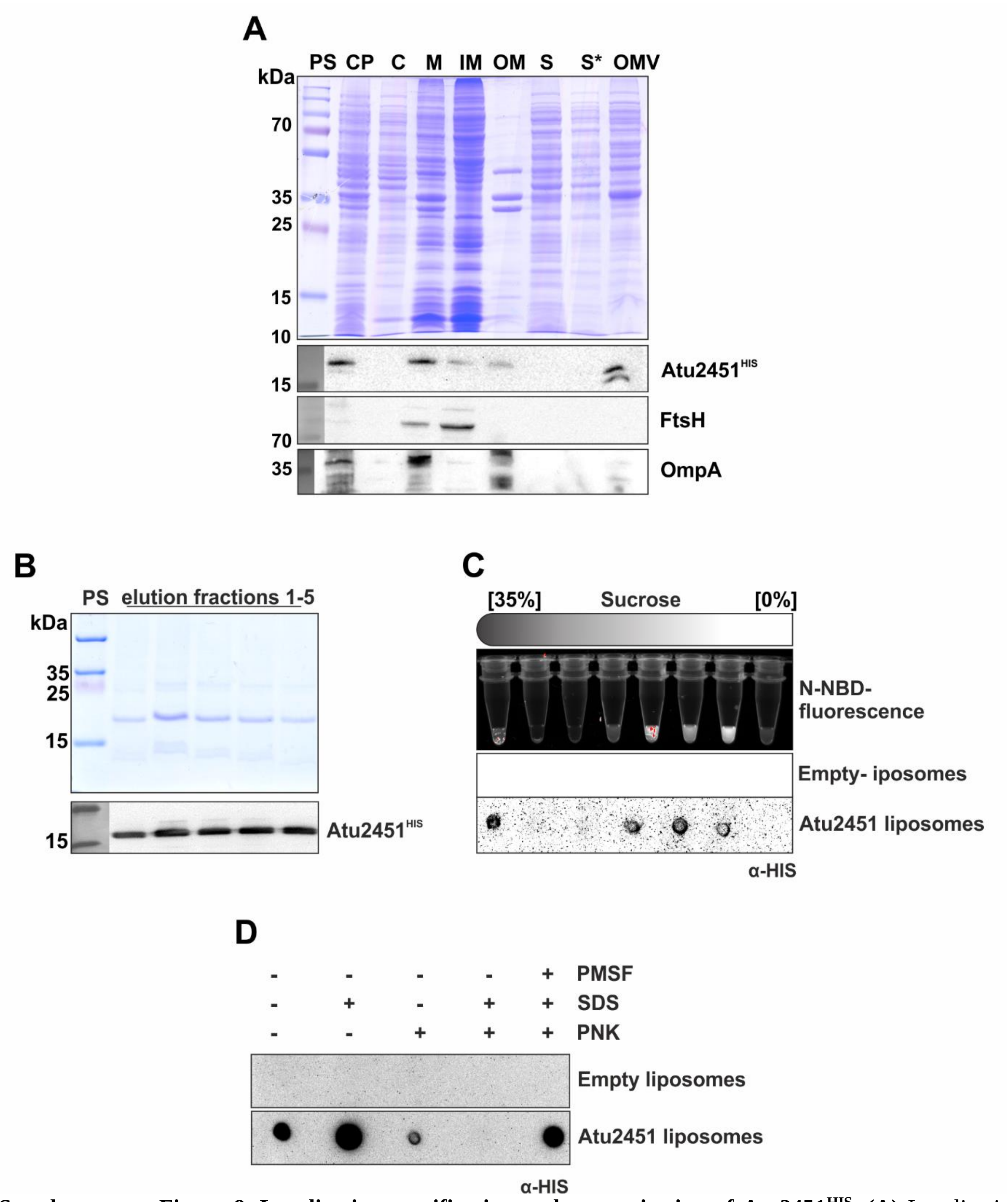

Supplementary Figure 8. Localization, purification and reconstitution of Atu2451^{HIS}. (A) Localization of Atu2451^{HIS} in *E. coli*. *E. coli* BL21 containing the pET_atu2451 expression plasmid was cultivated in LB medium at 37°C to an OD_{580} of 0.6. After induction with 0.4 mM IPTG, cells were cultivated for 4 h at 30°C. Cells were then harvested, disrupted and separated into cytosolic (C) and membrane fraction (M) by ultracentrifugation. The membrane fraction was treated with *N*-lauroylsarcosine for separation into inner and outer membranes (IM/OM). For the extracellular localization of proteins, the cell-free culture supernatant (S) was fractionated into secreted soluble proteins (S*) and OMV-associated proteins (OMV) by ultracentrifugation. Atu2451 was detected with a HIS-epitope specific antibody. FtsH served as inner membrane reference protein and OmpA as outer membrane control protein. Both proteins were detected with specific antibodies. **(B)** SDS-PAGE and Western blot analysis of Ni-IDA purified Atu2451^{HIS} from *E. coli* expression strains. **(C)** Efficiency of Atu2451^{HIS} reconstitution in liposomes containing the reporter lipid *N*-NBD-PE was analyzed by a flotation assay of Atu2451^{HIS} proteoliposomes in a sucrose gradient. Fractions were analyzed for NBD fluorescence (lipid marker) and protein content (Dot blot using anti-HIS antibodies). Reconstitution was evident from co-migration of phospholipid and Atu2451^{HIS}. **(D)** Proteinase K (PNK)-protection assay with reconstituted liposomes. A Dot blot using anti-HIS antibodies was conducted to detect Atu2451^{HIS} in intact or SDS-treated liposomes.

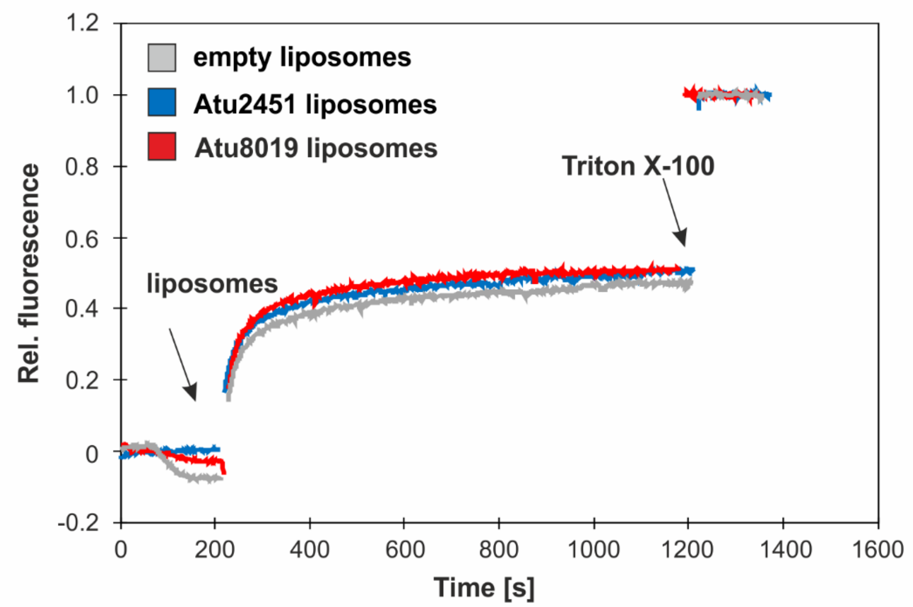

Supplementary Figure 9. Atu8019^{HIS} proteoliposomes do not disrupt artificial liposomes. Liposome leakage assay with DOPC liposomes containing self-quenching amounts of fluorescein in the presence of different (proteo)liposomes. To prepare liposomes with self-quenching amounts of fluorescein, lipids (100 µg DOPC) were dried under N_2 flow and rehydrated in the presence of 200 mM fluorescein (in 500 µL lysis buffer). After rehydration, liposomes were extruded through a 400 nm membrane and free dye was extracted using a PD10 column. Baseline fluorescence of 10 µL fluorescein-containing liposomes was recorded (λ_{Ex}= 488 nm, λ_{Ex}= 525 nm, 960 µL TBS buffer (50 mM Tris, 150 mM NaCl, pH 8.0)), before 30 µL Atu8019, Atu2451 or empty liposomes were added. Fluorescence was recorded over 1000 s, until signal stabilized, before 0.5% Triton X-100 were added to disrupt vesicles. Fluorescence signal was normalized to the baseline fluorescence, to obtain relative fluorescence.

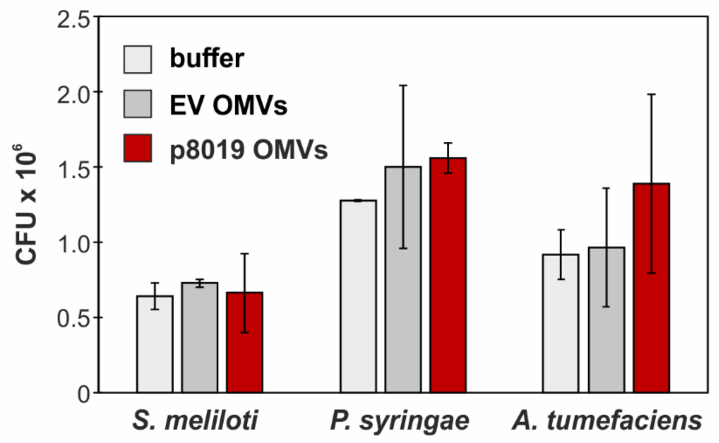

Supplementary Figure 10. **Atu8019-enriched OMVs have no inhibitory effect on target bacteria.** A mixture of target bacteria and OMVs derived from *A. tumefaciens* WT containing the empty vector (EV OMVs) or *atu8019* overexpression (p8019 OMVs) strain was prepared as described in Fig. 5. The mixture was incubated at RT for 2 h in TBS buffer. Cells incubated with TBS buffer served as negative control. 100 µL of dilutions (10^{-4} and 10^{-5}) were plated on LB-agar plates (*A. tumefaciens*, *P. syringae*) or TY-agar plates (*S. meliloti*). After incubation for 3 days at 30°C, colony forming units were determined. Values are averages of duplicate assays and error bars are standard deviations.

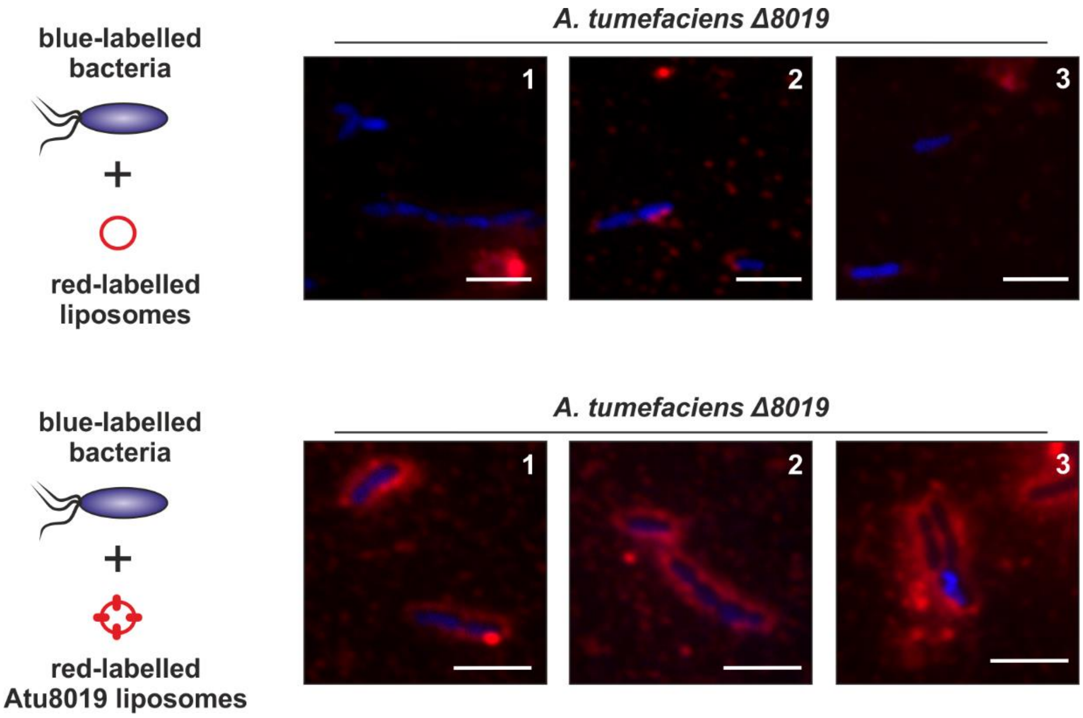

Supplementary Figure 11. Atu8019^{HIS} proteoliposomes interact with *A. tumefaciens atu8019* deletion strain. Interaction of empty liposomes and Atu8019 proteoliposomes with the *atu8019* deletion strain. Experiment was performed as described in Fig. 8. Scale bars: 5 µM.

Supplementary Tables

Supplementary Table 1. Strains and plasmids used in this study.

Strain	Relevant characteristic/description[a]	Reference
E. coli strains		
JM83	Cloning host	(Vieira & Messing, 1982)
BL21 (DE3)	Expression of recombinant proteins	(Studier & Moffatt, 1986)
DH10B	Served as target bacterium	Stock collection Bochum, Germany
A. tumefaciens strains		
C58	WT, pTiC58, pAtC58	C. Baron
C58 Δ*atu8019*	WT-derivative with *atu8019* deletion	This study
C58 *atu8019*^{FLAG}	WT-derivative, chromosomal integration of 3xFLAG-tag sequence at *atu8019* 3' end	This study
C58 *hfq*^{3xFLAG}	Chromosomal integration of 3xFLAG-tag sequence at *hfq* 3' end	(Möller *et al.*, 2014)
**Other strains**		
Sinorhizobium meliloti SM11	WT, served as target bacterium	Stock collection Bochum, Germany
Pseudomonas syringae pv. tomato DC3000	WT, Rif^R, served as target bacterium	Stock collection Bochum, Germany
Xanthomonas capmestris pv. campestris 8004	Wild-type; Rif^R, served as target bacterium	U. Bonas, Halle-Wittenberg, Germany
Bacillus subtilis	WT, *trpC*2, served as target bacterium	(Rahmer *et al.*, 2015)
**Plasmids**		
pET24b	Overproduction of HIS-tagged proteins, Km^R	Novagen, Madison, USA
pTrc200	expression vector, pVS1 origin, *lacI*$_q$, Strep^R, Spec^R, trc promoter	(Schmidt-Eisenlohr *et al.*, 1999)
pK19mobsac	suicide vector for mutagenesis, Km^R	(Schäfer *et al.*, 1994)
pYP168	pUC18 derivative with reduced MCS (SmaI only), Amp^R	(Hoffmann *et al.*, 2015)
pYP247	pYP190 derivative carrying FLAG-KaT; Km^R oriT	(Hoffmann *et al.*, 2015)
pBO6100	pK19mobsacB derivative carrying the up- and downstream regions of *atu8019*	This study
pBO6101	pYPUB168 derivative carrying *atu8019*	This study
pBO6102	pYP168 derivative carrying *atu8019*-FLAG; Km^R oriT	This study
pBO6104	Trc200 derivative encoding Atu8019C22A	This study
pBO6121	pTrc200 derivative encoding tagless Atu8019	This study
pBO6126	pET24b derivative carrying *atu8019*	This study
pBO6127	pET24b derivative encoding Atu8019C22A	This study
pBO6128	pTrc200 derivative carrying *atu8019*	This study
pEX_8019_syn	pEXA2 derivative carrying *atu8019*	Eurofins Genomics,
pBO1920	pET24b derivative carrying *atu2451*	This study

[a]Amp, ampicillin; Km, kanamycin; Spec, spectinomycin; Strep, streptomycin Rif, rifampicin

Supplementary Table 2. Oligonucleotides used in this study.

Primer	Oligonucleotide sequence (5`→3`)[a]
**pTrc200-*atu8019* expression plasmids**	
8019-Nco-fw	AAACCATGGCUATCACACGTATTCTTTCG
8019-Bam-rv	AAAGGATCCTCAGTGGTGGTGGTGGTG
8019-rev-mS	AAAGGATCCTTAGCGGCGTGCGGC
**pET24b-*atu2451* expression plasmid**	
2451-Nde-fw	AAACATATGCGTGGCGTATTTGCCGT
2451-Xho-rv	AAACTCGAGGCGATATCCGCACTGCTG
**Site-directed mutagenesis of *atu8019***	
8019C22A-fw	GTCACCCTCAGTTCC**GCG**GGCAACACCATTC
8019C22A-rv	GAATGGTGTTGCC**CGC**GGAACTGAGGGTGAC
**Deletion mutagenesis**	
8019UP-Eco-fw	AAAGAATTCAGGTAAATTGCGTGATG
8019UP-Pst-rv	AAACTGCAGGTGAAACTACTCCCCTATG
8019DWN-Pst-fw	AAACTGCAGTAATCAAGGCTCCCCTC
8019DWN-Hind-rv	AAAAAGCTTAACGGGCGAATAGTCG
**FLAG-reporter strain**	
8019flag-fw	AAGCTTGCGCCACCTGCCGTCATCACG
8019flag-rv	GGGGCGGCGTGCGGCGCG
**pPCR**	
3xflagfw_bamhI	AAAAGGATCCGACTACAAAGACCATGACGGTG
3xflagrv_acc65I	AAAAGGTACCTCATTTATCGTCGTCATCTTTGTAG
chvE-fw	GCTGTCCCAGATCGAAAA
chvE-rev	GCCTGCTTCAGAACGTC

[a] Restriction sites are underlined and bp-exchanges are shown in bold.

Supplementary Table 3. List of proteins identified by mass spectrometry in OMVs isolated from *A. tumefaciens* C58 grown in LB medium. The number (#) of replicates in which the proteins were identified with at least one peptide is given. Proteins identified in more than one replicate (gray) and Atu2451 (light blue) and Atu8019 (red) are highlighted.

Protein identifier			Description	# of replicates
Atu0048		AAK85872	YkuD domain-containing protein	2
Atu0084	RpsO	AAK85904	30S ribosomal protein S15	2
Atu0224	CtpA	AAK86042	components of type IV pilus pilin subunit	1
Atu0256	RplT	AAK86072	50S ribosomal protein L20	1
Atu0542	Fla	AAK86355	flagellin	3
Atu0543	FlaB	AAK86356	flagellin	3
Atu0545	FlaA	AAK86357	flagellin	3
Atu0552	FlgG	AAK86364	flagellar basal body rod protein	2
Atu0567	FlaD	AAK86378	flagellin	3
Atu0574	FlgE	AAK86385	flagellar hook protein	3
Atu0575	FlgK	AAK86386	flagellar hook associated protein	3
Atu0576	FlgL	AAK86387	flagellin	3
Atu0584		AAK86395	Rod-binding domain-containing protein	1
Atu0650		AAK86458	uncharacterized protein	3
Atu0653		AAK86461	uncharacterized protein	2
Atu0682	GroL	AAK86491	60 kDa chaperonin	3
Atu0688	TadA	AAK86497	tRNA-specific adenosine deaminase	1
Atu0782		AAK86591	UPF0337 protein	1
Atu0844		AAK86651	YkuD domain-containing protein	1
Atu0845		AAK86652	YkuD domain-containing protein	2
Atu1020		AAK86828	porin	3
Atu1021		AAK86830	porin	3
Atu1090	RpsR	AAL42103	30S ribosomal protein S18	1
Atu1091	RpsF	AAL42104	30S ribosomal protein S6	1
Atu1133	RopB	AAK86934	outer membrane protein	3
Atu1246	RpsI	AAK87043	30S ribosomal protein S9	2
Atu1247	RplM	AAK87044	50S ribosomal protein L13	1
Atu1262	HupA	AAK87057	histone like protein	1
Atu1374	RpsB	AAK87166	30S ribosomal protein S2	1
Atu1426	Eno	AAK87218	enolase	2
Atu1525		AAK87316	uncharacterized protein	1
Atu1577		AAK87360	ABC transporter substrate binding protein amino acid	2
Atu1664	Tig	AAK87437	trigger factor	2
Atu1717	FadL	AAK87490	long chain fatty acid transport protein	2
Atu1770	GlnA	AAK87539	glutamine synthetase	2
Atu1846		AAK87613	uncharacterized protein	1
Atu1860	RpsD	AAK87627	30S ribosomal protein S4	2
Atu1922	RplQ	AAK87683	50S ribosomal protein L17	1
Atu1925	RpsM	AAK87686	30S ribosomal protein S13	1
Atu1928	RplO	AAK87690	50S ribosomal protein L15	1
Atu1929	RpsE	AAK87692	30S ribosomal protein S5	2
Atu1930	RplR	AAK87693	50S ribosomal protein L18	3
Atu1932	RpsH	AAK87695	30S ribosomal protein S8	2
Atu1936	RplN	AAK87699	50S ribosomal protein L14	1
Atu1937	RpsQ	AAL42933	30S ribosomal protein S17	1
Atu1939	RplP	AAK87701	50S ribosomal protein L16	1
Atu1940	RpsC	AAK87702	30S ribosomal protein S3	1

Protein identifier			Description	# of replicates
Atu1941	RplV	AAK87703	50S ribosomal protein L22	1
Atu1942	RpsS	AAK87704	30S ribosomal protein S19	1
Atu1943	RplB	AAK87705	50S ribosomal protein L2	1
Atu1944	RplW	AAK87706	50S ribosomal protein L23	1
Atu1945		AAK87707	50S ribosomal protein L4	2
Atu1946	RplC	AAK87708	50S ribosomal protein L3	1
Atu1947	RpsJ	AAL42943	30S ribosomal protein S10	1
Atu1948	TufA	AAK87709	elongation factor TU	2
Atu1950	RpsG	AAK87711	30S ribosomal protein S7	1
Atu1951	RpsL	AAK87712	30S ribosomal protein S12	1
Atu1958	RplJ	AAK87718	50S ribosomal Protein L10	2
Atu1959	RplA	AAK87719	50S ribosomal protein L1	1
Atu1960	RplK	AAK87720	50S Ribosomal Protein L11	1
Atu2084	BamD	AAK87834	outer membrane protein assembly factor	2
Atu2159	Omp	AAK87905	outer membrane protein	2
Atu2227	RplY	AAK87969	50S ribosomal protein L25	1
Atu2439		AAK88176	uncharacterized protein	1
Atu2451		AAK88188	SLT domain-containing protein	3
Atu2614		AAK88336	gly-zipper_Omp domain-containing protein	2
Atu2638	SucC	AAK88359	succinyl CoA synthetase beta chain	1
Atu2641	Omp19	AAK88363	outer membrane lipoprotein	2
Atu2699	RpsP	AAK88419	30S ribosomal protein S16	1
Atu2703	RplS	AAK88423	50S ribosomal protein L19	2
Atu2722	OmpA	AAK88440	porin	2
Atu2784	RpmA	AAK88499	50S ribosomal protein L27	1
Atu2787		AAL43768	DUF1737 domain-containing protein	1
Atu3052		AAK90330	transcriptional regulator MarR family	1
Atu3091		AAK90292	uncharacterized protein	2
Atu3191		AAK90195	outer membrane protein	2
Atu3331		AAK90060	YkuD domain-containing protein	2
Atu3642		AAK89755	VgrG protein	1
Atu3708	RplD	AAK89697	outer surface protein	2
Atu3713	PalA	AAK89692	peptidoglycan-associated lipoprotein	3
Atu3714	TolB	AAK89691	Tol-Pal system protein TolB	2
Atu3737	GapA	AAK89669	glyceraldehyde 3 Phosphate Dehydrogenase	2
Atu4026		AAK89401	uncharacterized protein	2
Atu4063		AAK89365	O-linked GlcNAc transferase	2
Atu4064	RpsU1	AAK89364	30S ribosomal protein S21	1
Atu4086	IdnO	AAK89345	gluconate 5 dehydrogenase	1
Atu4345		AAK89095	uncharacterized protein	3
Atu4347		AAK89093	uncharacterized protein	3
Atu4348	VgrG	AAK89092	VgrG protein	1
Atu4350		AAK89090	Ntox15 domain-containing protein	1
Atu4587		AAK88858	uncharacterized protein	1
Atu4669		AAK88778	uncharacterized protein	3
Atu5118		AAK90492	aminotransferase class II	1
Atu5121		AAK90495	two component response regulator	1
Atu5170	AvhB9	AAK90541	type IV secretion protein AvhB9	1
Atu5283		AAK90659	ECF family sigma factor	1
Atu5296		AAK90671	arylester hydrolase	2
Atu5483		AAK90860	transcriptional regulator GntR family	1
Atu8019		ABW89715	uncharacterized protein	3

Protein identifier			Description	# of replicates
Atu8122		AAK86250	uncharacterized protein	3

Supplementary Table 4. Details for MS-based identification of Atu8019 and Atu2451 in OMVs isolated from *A. tumefaciens* C58 grown in LB medium. For each of three independent biological replicates, the protein score derived from ProteinLynxGlobalServer (PLGS, Waters), the sequence coverage, the number of identified and theoretical tryptic peptides as well as the number of found fragment ions are given.

Protein	Replicate	Protein Score	Sequence Coverage [%]	Identified Peptides	Theoretical Peptides	Fragment Ions
Atu8019	1	19385	26.42	1	3	19
	2	2496	33.96	2		16
	3	12055	26.42	1		14
Atu2451	1	3952	30.41	7	14	37
	2	228	26.80	5		18
	3	9487	39.69	6		48

Supplementary Table 5. Tryptic peptides identified for Atu8019. For three biological replicates, tryptic peptides identified by MS are highlighted in the protein sequence of Atu8019 (53 amino acids).

Replicate	Peptide	Atu8019 Sequence
1	1	1-MITRILSTVFVALLTVVTLSSCGNTIRGVGR**DTANAVDATQDAGR**SVDRAARR-53
2	1	1-MITRILSTVFVALLTVVTLSSCGNTIRGVGR**DTANAVDATQDAGR**SVDRAARR-53
	2	1-MITRILSTVFVALLTVVTLSSCGNTIR**GVGRDTANAVDATQDAGR**SVDRAARR-53
3	1	1-MITRILSTVFVALLTVVTLSSCGNTIRGVGR**DTANAVDATQDAGR**SVDRAARR-53

Supplementary References

Hoffmann, M.C., Wagner, E., Langklotz, S., Pfänder, Y., Hott, S., Bandow, J.E., and Masepohl, B. (2015) Proteome profiling of the *Rhodobacter capsulatus* molybdenum response reveals a role of IscN in nitrogen fixation by Fe-nitrogenase. *J Bacteriol* **198**: 633-643.

Möller, P., Overlöper, A., Forstner, K.U., Wen, T.N., Sharma, C.M., Lai, E.M., and Narberhaus, F. (2014) Profound impact of Hfq on nutrient acquisition, metabolism and motility in the plant pathogen *Agrobacterium tumefaciens*. *PLoS One* **9**: doi: 10.1371/journal.pone.0110427.

Rahmer, R., Morabbi Heravi, K., and Altenbuchner, J. (2015) Construction of a super-competent *Bacillus subtilis* 168 using the P_{mtlA} -*comKS* inducible cassette. *Front Microbiol* **6**: doi: 10.3389/fmicb.2015.01431.

Schäfer, A., Tauch, A., Jager, W., Kalinowski, J., Thierbach, G., and Pühler, A. (1994) Small mobilizable multi-purpose cloning vectors derived from the *Escherichia coli* plasmids pK18 and pK19: selection of defined deletions in the chromosome of *Corynebacterium glutamicum*. *Gene* **145**: 69-73.

Schmidt-Eisenlohr, H., Domke, N., Angerer, C., Wanner, G., Zambryski, P.C., and Baron, C. (1999) Vir proteins stabilize VirB5 and mediate its association with the T pilus of *Agrobacterium tumefaciens*. *J Bacteriol* **181**: 7485-7492.

Studier, F.W., and Moffatt, B.A. (1986) Use of bacteriophage T7 RNA polymerase to direct selective high-level expression of cloned genes. *J Mol Biol* **189**: 113-130.

Vieira, J., and Messing, J. (1982) The pUC plasmids, an M13mp7-derived system for insertion mutagenesis and sequencing with synthetic universal primers. *Gene* **19**: 259-268.

Wu, H.Y., Liu, K.H., Wang, Y.C., Wu, J.F., Chiu, W.L., Chen, C.Y., Wu, S.H., Sheen, J., and Lai, E.M. (2014) AGROBEST: an efficient *Agrobacterium-mediated* transient expression method for versatile gene function analyses in *Arabidopsis* seedlings. *Plant Methods* **10**: doi: 10.1186/1746-4811-1110-1119.
